# Microbiome Heritability and Its Role in Adaptation of Hosts to Novel Resources

**DOI:** 10.3389/fmicb.2022.703183

**Published:** 2022-07-05

**Authors:** Karen Bisschop, Hylke H. Kortenbosch, Timo J. B. van Eldijk, Cyrus A. Mallon, Joana F. Salles, Dries Bonte, Rampal S. Etienne

**Affiliations:** ^1^Groningen Institute for Evolutionary Life Sciences, University of Groningen, Groningen, Netherlands; ^2^Terrestrial Ecology Unit (TEREC), Department of Biology, Ghent University, Ghent, Belgium; ^3^Institute for Biodiversity and Ecosystem Dynamics, University of Amsterdam, Amsterdam, Netherlands; ^4^Laboratory of Aquatic Biology, Department of Biology, KU Leuven, Kortrijk, Belgium

**Keywords:** local adaptation, bacterial communities, endosymbionts, spider mites, *Tetranychus urticae*

## Abstract

Microbiomes are involved in most vital processes, such as immune response, detoxification, and digestion and are thereby elementary to organismal functioning and ultimately the host’s fitness. In turn, the microbiome may be influenced by the host and by the host’s environment. To understand microbiome dynamics during the process of adaptation to new resources, we performed an evolutionary experiment with the two-spotted spider mite, *Tetranychus urticae*. We generated genetically depleted strains of the two-spotted spider mite and reared them on their ancestral host plant and two novel host plants for approximately 12 generations. The use of genetically depleted strains reduced the magnitude of genetic adaptation of the spider mite host to the new resource and, hence, allowed for better detection of signals of adaptation *via* the microbiome. During the course of adaptation, we tested spider mite performance (number of eggs laid and longevity) and characterized the bacterial component of its microbiome (16S rRNA gene sequencing) to determine: (1) whether the bacterial communities were shaped by mite ancestry or plant environment and (2) whether the spider mites’ performance and microbiome composition were related. We found that spider mite performance on the novel host plants was clearly correlated with microbiome composition. Because our results show that only little of the total variation in the microbiome can be explained by the properties of the host (spider mite) and the environment (plant species) we studied, we argue that the bacterial community within hosts could be valuable for understanding a species’ performance on multiple resources.

## Introduction

Microbiomes are communities of microorganisms and their associated gene expression in a particular environment (Berg et al., 2020). Hence, the microbiome of a certain host species includes all microorganisms on the inside and outside of an organism with no distinction between microbes with beneficial, neutral, or detrimental effects on their host. Over the last decade, research on host-microbiome interactions has revealed that microbial communities can influence host immunity, digestion and detoxification (Stecher and Hardt, 2008; Berendsen et al., 2012; Huttenhower et al., 2012; David et al., 2014; Kohl et al., 2014; Kohl and Dearing, 2016). These complex, often beneficial host-microbiome interactions illustrate the importance of microbiota in their ability to affect the performance of their hosts. For instance, Zhu et al. (2019) showed that lower fecundity was associated with lower bacterial diversity in the cassava mite, *Tetranychus truncatus*. The importance of the microbiome for host phenotype has led some to suggest the concept of the “holobiont” (i.e., the host with its microorganisms) as a unit of selection (Zilber-Rosenberg and Rosenberg, 2008; Theis et al., 2016). A single unit of selection implies, however, one interconnected fate of the host and his microbiome which has led to a lot of criticism as microbiomes are rarely entirely inherited (Moran and Sloan, 2015; Henry et al., 2021). The transmission occurs *via* vertical transfer from parent to offspring, horizontal transfer from the environment, or both (Ebert, 2013; Henry et al., 2021). Therefore, some prefer the “extended genotype” where the microorganisms are having extended effects on the host phenotype and may shift the mean host phenotype or change the phenotypic variance in a population (Henry et al., 2021).

Because microbiomes are usually diverse, the versatility of different bacteria may help for better functioning of the host and assist in optimal adaptation to changing conditions (Zilber-Rosenberg and Rosenberg, 2008). It has been found that the bacterial communities need to be specialized to improve the adaptive fitness of the host, the influence of more homogeneous communities is often negligible (Huitzil et al., 2018). For herbivores adapting to novel resources, the microbiome may assist in the digestion of cellulose or lignin and the detoxification of potential poisonous substances (David et al., 2014; Franchini et al., 2014; Kohl et al., 2014; Kohl and Dearing, 2016; Staudacher et al., 2017). Microbial symbionts may even contribute to the synthesis of essential nutrients as seen in aphids where the obligate symbiont *Buchnera aphidicola* provides missing amino acids (Baumann, 2005; Oliver et al., 2010). Novel resources often cause other challenges such as parasitoids or fungi, however, a diverse community of facultative symbionts may provide protection against those challenges (Oliver et al., 2010; Vorburger and Perlman, 2018; Hafer and Vorburger, 2019). For instance, some aphid populations showed improved fecundity when feeding on clover if they were infected with *Regiella insecticola* (Leonardo and Muiru, 2003; Oliver et al., 2010; Zélé et al., 2018a). Besides beneficial host-microbiome interactions, some facultative symbionts are also known to interfere with reproduction, such as *Wolbachia*, *Cardinium*, and *Spiroplasma* spp. (Breeuwer and Jacobs, 1996; Gotoh et al., 2003, 2007; Enigl and Schausberger, 2007; Xie et al., 2016). These endosymbionts use cytoplasmic incompatibility, feminization, parthenogenesis or male killing to secure their persistence (Staudacher et al., 2017). However, it remains unclear whether only these endosymbionts rather than the microbiome as a whole are responsible for these effects (Brinker et al., 2019).

Host-microbiome interactions are not unidirectional and microbiomes themselves are known to be affected by host diet, host taxonomy and host genetics (Santo Domingo et al., 1998; Broderick et al., 2004; Spor et al., 2011; Colman et al., 2012; Jandhyala et al., 2015). A study on whiteflies suggested that the host’s genome influences the potential fitness benefits of *Rickettsia* (Cass et al., 2016), as *Rickettsia* decreased the developmental time and increased the fecundity in one but not another genetic line of whiteflies. Another example was provided by Chaplinska et al., 2016 who showed the importance of the population background (i.e., genotype, geographic origin, and founder effects) of the host. They discovered differences in microbiome composition between different *Drosophila melanogaster* populations that had been maintained on the same food source and laboratory conditions for several years. However, because these populations had never been mixed, it was difficult to disentangle founder effects and drift from selection. Nonetheless, the fact that different microbiomes could persist and were transgenerationally transmitted shows that some components of the microbiome are heritable (Chaplinska et al., 2016).

The host-microbiome concepts (Bourtzis et al., 1996; Bordenstein et al., 2001; Shin et al., 2011; Sommer and Bäckhed, 2013; Lizé et al., 2014; Chaplinska et al., 2016) developed for insects can be extended to other arthropods such as the two-spotted spider mite or *Tetranychus urticae*, the focal species in this study. It has, for instance, been found in aphids that endosymbionts can offer protection against fungi or parasitoids (Oliver et al., 2005, 2010; Scarborough et al., 2005; Łukasik et al., 2013; Weldon et al., 2013; Guidolin et al., 2018; King, 2019), suggesting that endosymbionts may play a role in host immunity for other arthropods as well. So far, mainly behavioral adaptation such as avoidance of contaminated food has been found in the two-spotted spider mite, but no resistance or tolerance mechanisms against pathogenic bacteria (Santos-Matos et al., 2017; Zélé et al., 2019), although the latter may be due to the fact that the most common endosymbionts in spider mites (*Wolbachia*, *Rickettsia*, *Cardinium*, and *Arsenophonus*) were not present in the studied spider mite populations (Santos-Matos et al., 2017). Additionally, many microbial genes have been discovered within the *T. urticae* genome. These genes have different biochemical functions, such as the potential biosynthesis of pantothenate or vitamin B_5_ (Wybouw et al., 2018). Given that this vitamin is essential for the biosynthesis of for instance fatty acids and peptides (Kleinkauf, 2000), this hints at a deep evolutionary significance of endosymbionts in *T. urticae* adaptation.

We here aimed to elucidate the role of the bacterial component of the microbiome of *T. urticae* in the host’s adaptation to novel resources (i.e., host plants) using experimental evolution. More precisely, we (1) investigate the heritability of the spider mite-associated bacterial communities by looking at the relative effects of host plant and spider mite line on the bacterial communities and (2) study the relationship between the performance of the spider mite host on the different plant species and the bacterial composition. Adaptation of the spider mite host in our study does not refer to genetic adaptation, but to an overall improvement in host performance, which could be explained by the host-associated bacterial communities as well. The use of the microbiome as a fast-response mechanisms to changes in the environment has also recently been suggested (Voolstra and Ziegler, 2020).

From a single ancestral population, we created genetically depleted lines of *T. urticae*. These lines originated from one generation of mother-son mating and were therefore not homozygous inbred lines. We also created mixed lines by placing individuals from different genetically depleted lines together. On the one hand, the use of genetically depleted lines minimizes the chance for genetic adaptation (due to lower genetic variation) and increases the possibility to detect signals of adaptation *via* the microbiome, while on the other hand, the increase in genetic variation in the mixed lines will enhance the opportunity for genetic adaptation. These lines were transferred onto their initial host plant and two challenging novel resources for 150 days (about 12 mite generations). Performance on the host plants was recorded at several time points and the bacterial component of the mite microbiome was assessed after 150 days *via* high throughput sequencing of the V3-V4 region of the 16S rRNA gene.

While we only found a minor role for spider mite ancestry and host plant in determining the bacterial component of the mite microbiome, we report a substantial correlation between the total performance (i.e., fecundity and longevity) of the spider mite lines on the novel resources and the composition of bacterial communities associated with these lines.

## Materials and Methods

### Model System: Spider Mites and Plants

The two-spotted spider mite *Tetranychus urticae* Koch, 1836, is a model organism that is widely used in evolutionary experiments due to its well-known biology, small body size, high fecundity and short generation time (Bitume et al., 2013; Magalhães et al., 2014; Rodrigues et al., 2016; Alzate et al., 2017, 2019; Bisschop et al., 2019). Here, we used a stock population that was created by assembling different inbred lines that were created by Bitume et al. (2013) in August 2015. The initial collection of the stock population dates back to October 2000 when mites were collected from roses near Ghent, Belgium. The stock population has always been maintained on bean plants, *Phaseolus vulgaris* Prelude, at 18:6 L:D (light dark cycle) and 25°C. We here tested adaptation to novel host plants, cucumber *Cucumis sativus* Marketmaker, and tomato *Solanum lycopersicum* Moneymaker. We used two-week-old bean plants, four-week-old cucumber plants and six-week-old tomato plants. The ages of the host plants were chosen based on a previously performed pilot study to provide similar amounts of resource per plant species to the spider mite populations. Plants were grown in controlled greenhouse conditions at 28°C under 12:12 L:D and watered three times a week.

### Creating Genetically Depleted Lines to Allow for Testing the Effect of Host Ancestry

We sampled 20 deutonymph females from the stock population in January 2017 and created genetically depleted spider mite lines by placing each deutonymph female separately on bean leaf cuts and fertilizing her with her own sons resulting in one mother-son mating (*T. urticae* only produced haploid male offspring as they did not mate before; Figure 1.1). We want to emphasize that this procedure was followed to reduce the impact of genetic variation on adaptation, and not to create entirely homozygous inbred lines. Out of these 20 lines, we selected the nine lines that were best growing after two generations and maintained them on bean plants at 25°C and 18:6 L:D. These nine lines were subjected to microsatellite analysis and bacterial community analysis using denaturing gradient gel electrophoresis (DGGE, Pereira Silva et al., 2012). The six most different lines, both in terms of their genome and bacterial community composition (see Supplementary Materials and Methods S1) were then selected to ensure sufficient genetic and microbial variation across lines (from here-on the different genetically depleted lines are numbered as lines 1 to 6).

**Figure 1:**
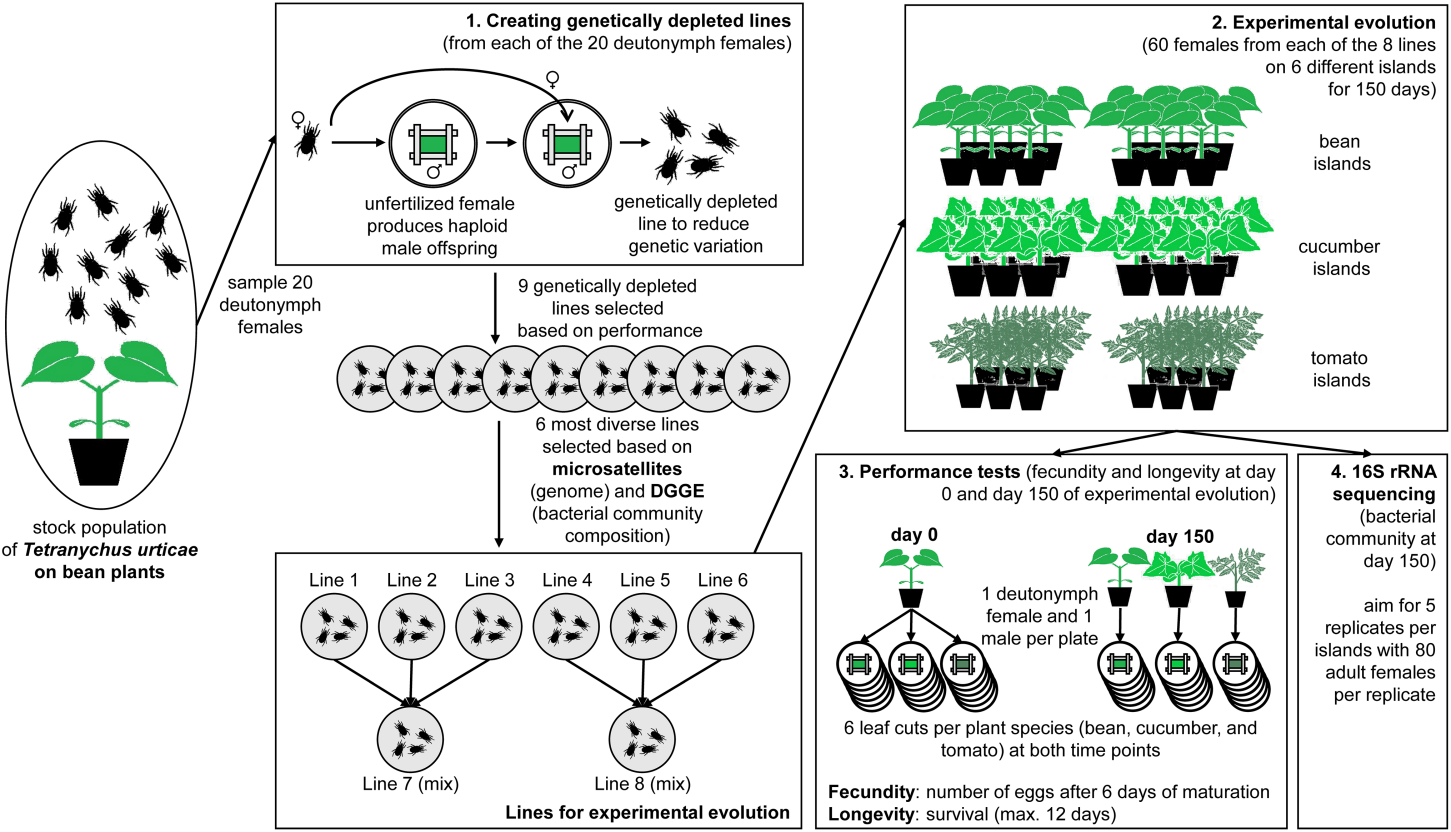
Overview figure of the performed experiment. The experiment started from a stock population of *Tetranychus urticae* on bean plants of which 20 deutonymph females were sampled to create genetically depleted lines. **(1)** The genetically depleted lines were derived from one mother-son mating from each of the 20 unfertilized female; the six most diverse lines were chosen for the experimental evolution (based on microsatellites and DGGE); three lines were combined for two mixed lines. **(2)** Each of the eight lines was subjected to experimental evolution on their initial host plants (two bean islands consisting of six plants each) and two novel host plants (two cucumber and two tomato islands consisting of six plants each) for 150 days. **(3)** Performance tests were performed after 0 and 150 days by transferring one deutonymph female and one male together to a leaf cut (six replicates) and counting the number of eggs after 6 days (i.e., fecundity) and the survival (i.e., longevity). **(4)** The bacterial component of the microbiome was measured *via* 16S rRNA sequencing after 150 days of experimental evolution.

### Design of Experiment: A Scenario for Local Adaptation

Each genetically depleted spider mite line was transferred to two bean “islands,” two cucumber “islands” and two tomato “islands” with an initial population size of 60 adult females on each island (Figure 1.2). These islands were groups of six plants maintained in separate open boxes with sticky paper at the bottom and Vaseline at the sides to prevent contamination between the populations. Besides the six original lines, two additional lines were created by mixing 20 adult females from lines 1, 2 and 3, and lines 4, 5 and 6, respectively (hereafter referred to as lines 7 and 8). This allowed us to study the effect of the level of standing genetic variation, because these mixed lines were genetically more diverse. For logistical reasons, the experiment was divided into two batches that were run 1 month apart from each other. The first batch contained lines 1, 2 and 3 and its mixed line 7; the second contained lines 4, 5 and 6 and its mixed line 8. This resulted in a total of 48 islands or 24 islands per batch (six islands per spider mite line and another six islands per mixed line) that were divided among three climate rooms under the same conditions (25°C under an 18:6 l:D photoperiod). Every week, two fresh plants were placed in the middle of the island, while the two oldest plants were removed. We chose this refreshment method to minimize selection for dispersive phenotypes and make sure that all older plants were touching the fresh ones.

### Testing Performance on Different Hosts Plants

The performance tests (measurement of the two fitness proxies: fecundity and longevity) were at the start (day 0) and at the end of the experiment (day 150; Figure 1.3). At the start, 18 quiescent female deutonymphs and 18 adult males per genetically depleted line were sampled from bean plants. Pairs consisting of one quiescent female deutonymph and one adult male were separately placed on six leaf cuts (2 × 3 cm; within a Petri dish with wet cotton and bordered by paper strips) from each of the three different plant species (bean, cucumber, and tomato plants). At the end of the experiment, the same procedure was followed, but pairs of quiescent female deutonymphs and adult males were only placed on leaf cuts from the same plant species as the experimental island they were collected from. These Petri dishes were kept under the same climatic conditions as the experimental islands (25°C and 18:6 L:D).

Fecundity was measured by counting the total number of eggs and larvae 6 days after maturation of the female (Alzate et al., 2017, 2019). In case the quiescent deutonymph female did not reach maturity, she was replaced by another quiescent deutonymph with a maximum of three replacements. Longevity was counted as the number of days the female was alive with a maximum of 12 days. This maximum was imposed because after 12 days the influence of the decay of the leaves on longevity might be too large. The adult females were checked daily and, when possible, saved from unnatural deaths such as drowning in the cotton. In the case of an unnatural death or the individual was still alive after 12 days, it was censored from the survival data: when this happened before the sixth day, the data points were excluded from the fecundity data (because fecundity was measured on the sixth day, later drowning did not affect the outcome). Due to the possibility of unnatural deaths or quiescent deutonymph females not reaching maturation, the number of replicates per time point and plant species was not equal among all genetically depleted lines. The largest discrepancy in number of replicates for performance was between plant species, for instance less replicates on tomato plants than on bean plants, which matched the difficulties of the novel host plants (overview in Supplementary Figure S1).

In many studies investigating genetic adaptation juvenile and maternal effects are standardized by placing the individuals for two generations under common garden (Magalhães et al., 2011; Kawecki et al., 2012). We deliberately did not do this, because two additional mite generations on a benign host plant (as is usually done, see Alzate et al., 2017, 2019; Bisschop et al., 2019) might disrupt the signal of the microbiome due to the very short microbial generation times. This makes it impossible, however, to distinguish maternal effects from microbiome effects.

### Investigating the Internal Microbiome (Sampling, DNA Extraction, Sequencing, and Data Processing)

At the end of the experiment (after 150 days), we sampled 80 adult females per replicate for the microbiome (which was necessary to get sufficient DNA), and we aimed to take five replicates per island (Figure 1.4). Sampling was done by sucking adult females from the plants onto a filter using a small vacuum pump. The mites were then transferred into an Eppendorf tube. The samples were directly frozen at −25°C. We intended to also collect samples at the start, but only have data from four genetically depleted lines, due to failed sampling (i.e., too small population sizes), DNA extractions, or low numbers of sequencing reads. A comparison between the initial and final samples for those four lines can be found in Supplementary Figure S2.

After sampling, the mites were surface-sterilized with ethanol: we submerged the mites in 0.5 ml 90% ethanol for 20 min, removed the ethanol, washed four times with 0.2 ml sterile distilled water (centrifuge in between washes for 1 min at 1,000 g). Then, the mites were crushed in the 0.1 ml of sterile distilled water leftover from the last washing step. The crushing was performed using sterile pestles, powered with a cordless pellet mixing motor. The crushed mites were then transferred into powerbead tubes from the DNeasy PowerSoil kit (Qiagen). DNA was extracted according to the manufacturer’s protocol with two additional steps: (1) to enhance crushing of bacterial cell walls, 0.25 gr of 0.1 mm glass beads were added to the powerbead tubes, and (2) the bead beating face was elongated to two times 10 min instead of two times 5 min.

The resulting DNA was quantified using the Quant-iT™ PicoGreen™ dsDNA Assay kit following the manufacturer’s protocol, in order to standardize the amount of DNA used in the following PCR protocol. From each sample, bacterial community composition was determined by using 2.5 ng DNA as the template in a PCR reaction targeting the V3-V4 region of the 16S rRNA gene; PCR protocol and primer sequences for the first PCR are given in Supplementary Materials and Methods S2. After the first PCR, the quantity of the DNA was estimated using the Eurogentec SmartLadder MW-1700-10 during gel electrophoresis (1% agarose gel at 100 V for 40 min); it was necessary to keep track of the quantity to ensure enough DNA for sequencing (35 μl of 30 ng/μl). In most cases, the requirements were not met after a first PCR and hence additional PCRs from the same initial sample were performed starting with the same initial amount of DNA (average was three PCRs per sample). We did not start the second PCR from the previous PCR product as that would amplify errors introduced by PCR. The samples of the different PCRs were pooled per sample and purified using the QIAquick PCR Purification kit (QIAGEN). Library preparation, Illumina MiSeq (2x250bp) sequencing and demultiplexing were performed by INRA Science and Impact (GeT-PlaGe platform of GenoToul, INRA Auzeville).

After demultiplexing, the data were preprocessed using QIIME2 version 2017.12, denoised, amplicon sequence variants (ASVs) determined with DADA2 (Callahan et al., 2016) and primers were trimmed (max. sequence lengths for forward and reverse reads were set at 300 bp). A MAFFT alignment (Katoh et al., 2017) of the ASV sequences was made, which was used for constructing a phylogenetic tree using FastTree (Price et al., 2009, 2010). We used the 97% identity GreenGenes 13_8 reference database (DeSantis et al., 2006) for 16S rRNA for a taxonomy table. The resulting sequence table, phylogenetic tree, and taxonomy table were merged into a phyloseq object in R. Singletons were removed from the samples and all datasets were rarefied before analyzing to an even depth of minimum 24,712 (based on the number of reads per sample and the rarefaction curves; Supplementary Figure S3). We used five different random seeds for the rarefaction to limit influences from sampling bias. This was especially necessary as many of the reads were absorbed by a single family, which created a long tail of rare diversity in our microbial community, and hence a potential sampling bias during rarefaction. At this point, 148 samples were present with ASV numbers between 1,126 and 1,148. More microbiome samples were available in the rarefied datasets for bean, than for cucumber and tomato, the numbers were 73, 46, and 29, respectively (an overview of the number of samples per line and plant species is given in Supplementary Table S1). The main reason for this discrepancy is the smaller population sizes on cucumber and tomato as well as the fact that certain lines did not survive on cucumber (line 1) and tomato (lines 1 and 3).

### Data Analysis

The analyses below are divided in three sections: (1) the exploration of the bacterial communities within the spider mites, i.e., host-associated bacterial communities (the influence of the spider mite line and plant species on the alpha and beta diversity of the bacterial communities), (2) the investigation of the performance tests which include fecundity and longevity of the spider mites themselves, and (3) the relation between the bacterial communities (both alpha and beta diversity) and the performance of the spider mites.

#### Host-Associated Bacterial Communities

##### Alpha Diversity of Bacterial Communities

To investigate whether alpha diversity of the bacterial communities differed per host spider mite line and plant species, we first calculated three different metrics of alpha diversity for each community: Faith’s phylogenetic diversity, Shannon diversity index, and species richness. We used GLMMs with a lognormal distribution for Faith’s phylogenetic diversity and the Shannon diversity index, and with a negative binomial distribution for species richness. The distribution was chosen based on a goodness-of-fit test using Akaike’s Information Criterion (AIC). The metric for alpha diversity was used as the dependent variable, while the host plant species, host spider mite line, and their interaction were the explanatory variables. The different islands nested within their respective batches were treated as a random variable for the Shannon diversity index and species richness. For Faith’s phylogenetic diversity, the nested random variable induced an overfitting of the model, as the variance was estimated to be zero (Magnusson et al., 2018). Hence, we only considered the island as a random variable for phylogenetic diversity. We performed model selection with stepwise removal of the non-significant variables. The pairwise comparisons were corrected for multiple comparisons with the Tukey method.

##### Bacterial Community Composition

We used permutational analysis of variance (PERMANOVA; Anderson, 2017; Anderson et al., 2017) to investigate which variable had the largest influence on the beta diversity of the bacterial communities; the host ancestry (different spider mite lines) or the environment (different host plants). To comply with the requirement of homogeneity of multivariate dispersions for each potential grouping variable (tested with betadisper), we were not able to use the abundance-weighted statistics (weighted UniFrac and Bray-Curtis distance) and we only used the unweighted UniFrac distance metric as input in the PERMANOVAs (Bray and Curtis, 1957; Lozupone et al., 2011).

The independent variables were the different spider mite lines, the host plants, and their interaction. The different islands were added as groups within which permutations were constrained (total of 1,000 permutations). We used PCoA plots to visualize the ordination. We furthermore calculated average microbiome dissimilarity values from this ordination by taking the average of the values in the distance matrix.

Furthermore, we investigated the similarities in community structure between the separate and mixed spider mite lines on the different plant species with the Cramer-von Mises test statistic using the libshuff method in mothur v. 1.45.0 (Schloss et al., 2009). This test statistic explores the likelihood of randomly obtaining the same structure. The distance matrices were constructed per batch and per island using the unweighted UniFrac distance metric. We used Bonferroni’s correction for multiple pairwise comparisons.

#### Performance, i.e., Fecundity and Longevity of the Spider Mites

To investigate differences in fitness proxies per host plant species and host spider mite line, the fecundity and longevity were analyzed with GLMMs (with Gaussian distribution) and Mixed Effects Cox Models, respectively. In both cases, the maximal model consisted of all combinations with the host plant species and host spider mite line. The random variables were the different islands nested within the batches. We selected the model *via* stepwise removal of non-significant variables. Pairwise comparisons were adjusted for multiple comparisons by using the Tukey method for fecundity and the Benjamini and Hochberg method for longevity.

#### Relation Between Performance and Microbiome Composition

The link between the performance and bacterial community composition was tested using a Procrustes and a Mantel test (with Pearson correlation method and 9,999 permutations). Distances between mite performance and the distance matrix generated for the bacterial communities per spider mite line (using the unweighted UniFrac metric) were used. This was done for each host plant species separately (i.e., bean, cucumber, and tomato) to rule out major differences based on host plant species. Two different distance metrics were used for the Procrustes and Mantel test: Euclidean distance and Manhattan distance. We performed the tests for the effect on fecundity and longevity simultaneously and for each of them separately (only fecundity or only longevity). For the performance, the mean fecundity and/or longevity was taken per spider mite line. For the matrix based on the bacterial component of the microbiome, the data was merged per line and rarefied based on the number of reads per sample and host plant species; in this way each sample received equal weight in the analyses. We additionally performed the analyses on island level (see results in Supplementary Table S2), but for seven islands we only had one or two samples from the bacterial community (six out of seven on tomato islands) which could create a bias in the results. Hence, we only present results on the level of spider mite line.

We also tested whether the different alpha diversity metrics were related to fecundity and longevity (no direct measurements were possible from the same individuals for bacterial communities and performance measurements). We used GLMMs with Gaussian distribution after transforming the data to obtain a normal distribution (an arc-sine, logarithmic, and Box-Cox transformation on the mean Faith’s phylogenetic diversity, Shannon diversity index, and species richness, respectively). The alpha metric was the dependent variable and the plant species, fecundity/longevity and their interaction as independent variables. The different spider mite lines were used as a random variable.

Statistical analyses were performed in R version 4.1.2 (2021-05-18) and the following R packages: phyloseq version 1.36.0 (Mcmurdie and Holmes, 2013), vegan version 2.5–7 (Oksanen et al., 2020), glmmTMB 1.1.1 (Brooks et al., 2017), emmeans 1.6.1 (Lenth, 2021), fitdistrplus 1.1-5 (Delignette-Muller, 2015), coxme 2.2–16 (Therneau, 2020), bestNormalize 1.8.0 (Peterson, 2019). The R code is made available as Supplementary Material.

## Results

### Host-Associated Bacterial Communities

#### General Overview

**Figure 2:**
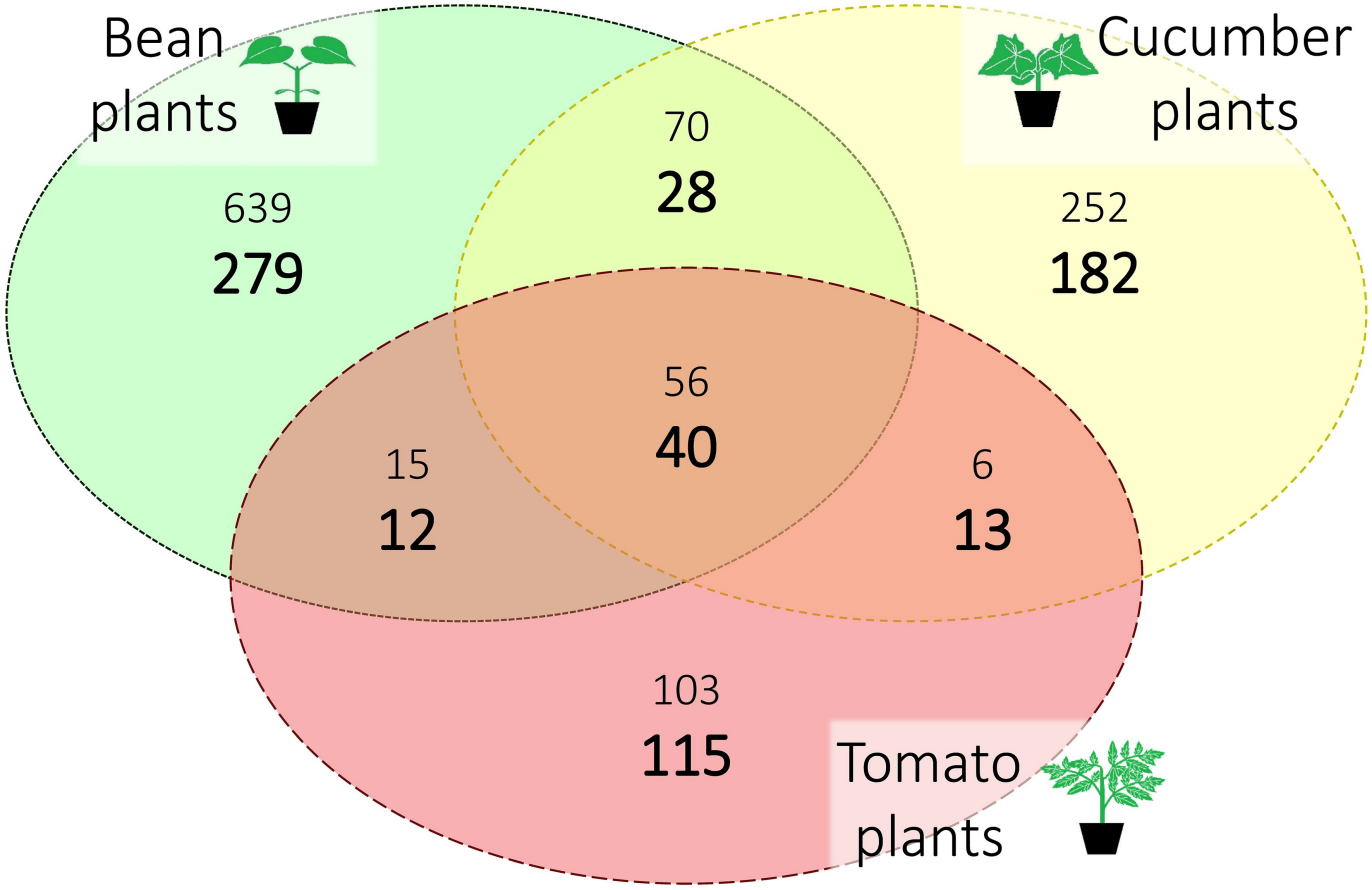
Distribution of the amplicon sequence variants (ASVs) over host plant species after rarefaction. The three different host plant species (bean, cucumber, and tomato) are represented with their unique and shared ASVs. Sample sizes per host plant were 73, 46, and 29 (for bean, cucumber, and tomato, respectively); the large, bold numbers are standardized for this sampling bias by selecting at random 29 samples (repeated 2,000 times) for each plant species. The smaller numbers are the absolute numbers of ASVs. All numbers are averaged over the seeds.

Out of the six spider mite lines created for this study, one line did not survive on tomato, and another line did not survive on both cucumber and tomato. For the surviving lines, we found between 1,126 and 1,148 ASVs with an average of 1,141 ASVs depending on the seed used during rarefaction. Most of this microbial diversity was found exclusively in the spider mite lines that had been put on bean plants, the ancestral host plant of the spider mite stock population. After correcting for the sampling bias (the number of samples from spider mite populations on bean, cucumber, and tomato were 73, 46, and 29, respectively; Supplementary Table S1) by selecting 29 samples per host plant species at random (and repeating this for 2,000 times), the average percentage of ASVs unique to bean, cucumber, and tomato were 41.7%, 27.2%, and 17.2%, respectively, while the average standardized percentage of ASVs common to all host plants was 6.0%. We provide an overview of the different host plants and their unique and shared ASVs in absolute and standardized numbers in Figure 2 (a complete overview of all ASVs per line and plant species is provided in Supplementary Table S3).

#### Alpha Diversity of Bacterial Communities

**Figure 3:**
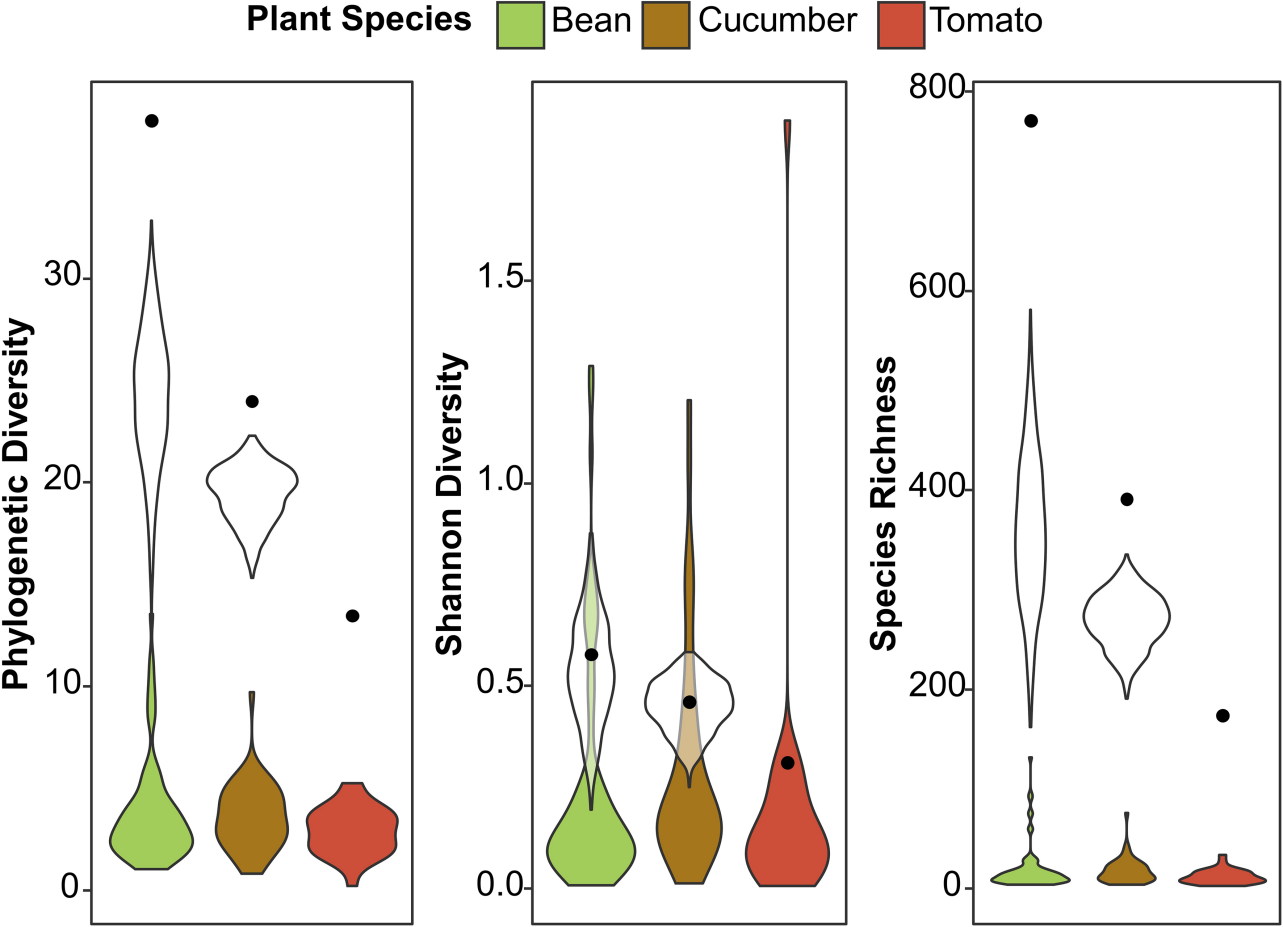
Alpha diversity among the different host plant species. The colored violin plots present the phylogenetic diversity, Shannon diversity, and species richness found per sample on the different host plant species. The black dots show the diversity from the total number of ASVs found in all the samples together (from 73, 46, and 29 samples for bean, cucumber, and tomato, respectively), while the white violin plots are standardized for the number of samples to prevent sampling bias (selection of 29 samples for each plant species, repeated 2,000 times). The black dots and white violin plots correspond to the absolute and standardized number of ASVs in Figure 1, respectively.

We used three diversity indices to quantify the alpha microbial diversity: Faith’s phylogenetic diversity, Shannon diversity, and species richness (Supplementary Figure S4; Supplementary Tables S4–S9). The results were consistent across the different random seeds used in rarefaction for microbial diversity. In general, spider mite line 5 had a higher alpha diversity than spider mite lines 1, 2, and 7 for Faith’s phylogenetic diversity and species richness. The spider mites’ host plant species could not explain the microbial variance found in alpha diversity and was not included in the most parsimonious model. Although the number of ASVs varied strongly between host plants, as reported above, we did not observe significant differences in the bacterial diversity between the samples from the different host plant species. We visualized this in Figure 3; where a large difference in species richness was found between host plant species when reads from all samples were pooled together, but, if individual samples were compared, the diversity did not differ among plant species. The mixed lines (line 7 and line 8) did not show a higher or lower microbial diversity than the genetically depleted lines, except for the significantly lower alpha diversity of line 7 compared to spider mite line 5 (Supplementary Figure S4).

#### Bacterial Community Composition

**Figure 4:**
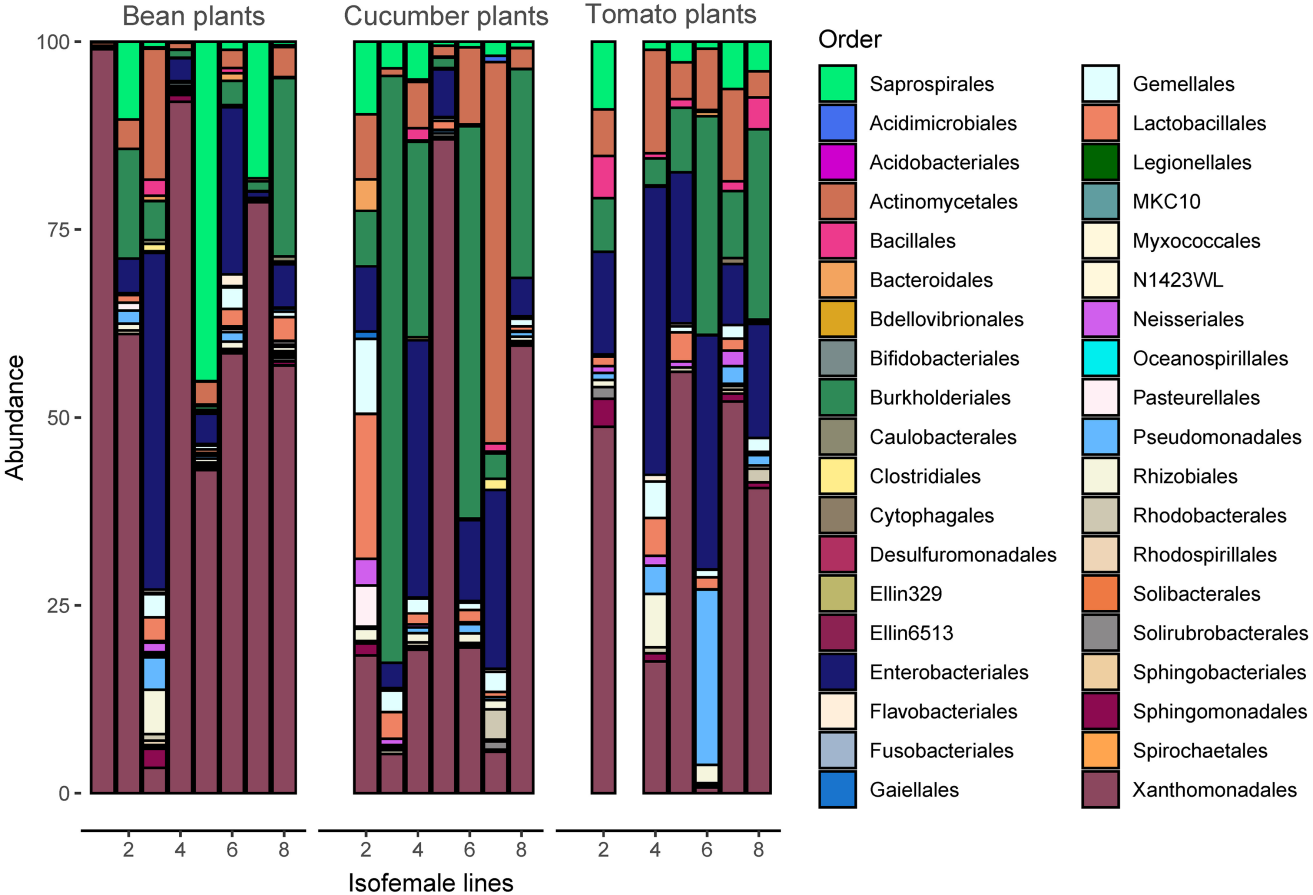
Taxonomy of the microbiome at the order level (the Rickettsiales are excluded from the figure for better visualization) per spider mite line and plant species. The most abundant taxonomic orders are Actinomycetales, Burkholderiales, Enterobacteriales, Saprospirales, and Xanthomonadales. A figure including Rickettsiales is provided in Supplementary Figure S5.

Analyses of bacterial community composition revealed a high abundance of Rickettsiales, mostly those belonging to genera *Wolbachia* and *Rickettsia* (Supplementary Figure S5). The significantly lower abundant orders that followed were the Xanthomonadales, Saprospirales, Enterobacteriales, Burkholderiales, and Actinomycetales (Figure 4). Despite the vast abundance of the Rickettsiales, they only comprised between 86 and 89 ASVs or between 7.6 and 7.8% (depending on the seed) of the total number of ASVs after rarefaction, indicating low genetic variability among Rickettsiales.

**Figure 5:**
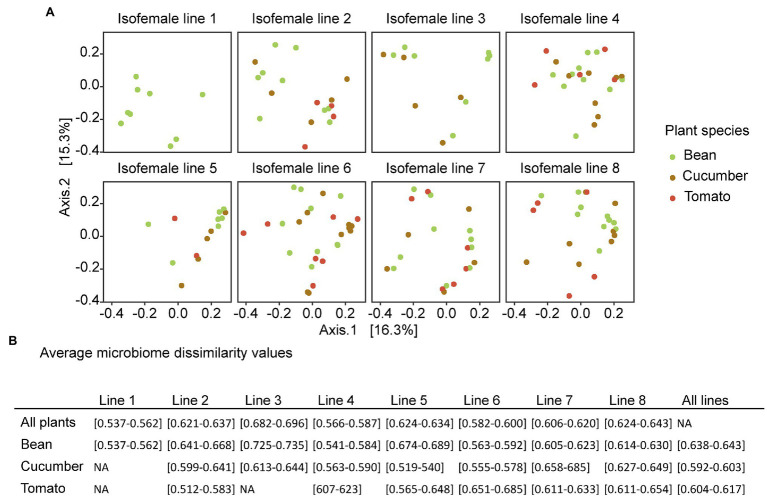
**(A)** PCoA plots based on the unweighted UniFrac, colored by plant species. The different plots represent the different spider mite lines under the same ordination. The PERMANOVA showed that only little of the variation is explained by the ancestral line and the host plant. Here, the result for one rarefied dataset is presented, but the plots from the other seeds used in rarefaction are in Supplementary Figure S6. **(B)** Average microbiome dissimilarity values per spider mite line based on the ordination of the PCoA plots. The results show the overall dissimilarity per spider mite line, the dissimilarity within a certain spider mite line and plant species, and the overall dissimilarity per host plant species. The interval includes all values for the different random seeds used in rarefaction.

The relatively low R-squared values from the PERMANOVA analyses indicated that a large amount of variation in the microbiomes of the spider mites remains unexplained by the tested variables (on average only 7.8%, 1.9%, and 6.7% for host ancestry, plant species, and their interaction, respectively, was explained, Table 1), which may explain the absence of clustering in the PCoA plot (Figure 5; Supplementary Figure S6). While for all data sets the host-associated bacterial communities were significantly determined by host ancestry, only one out of five seeds revealed significant results for plant species (Table 1).

**Table 1:** PERMANOVA output for the unweighted UniFrac.

Model: unweighted UniFrac ~ spider mite line * plant species (strata = batch)							
		Df	Sum of sqs	F.model	*R* ^2^	Pr(>F)	
Seed 1	**Line**	7	2.260	1.655	0.077	**0.001**	***
	Plant Species	2	0.571	1.465	0.019	0.063	
	Line: Plant Species	11	1.905	0.888	0.065	0.822	
	Residuals	127	24.765		0.839		
	Total	147	29.501		1.000		
Seed 2	**Line**	7	2.370	1.719	0.079	**0.001**	***
	Plant Species	2	0.597	1.513	0.020	**0.045**	*
	Line: Plant Species	11	2.058	0.949	0.068	0.650	
	Residuals	127	25.025		0.833		
	Total	147	30.050		1.000		
Seed 3	**Line**	7	2.345	1.723	0.079	**0.001**	***
	Plant Species	2	0.540	1.388	0.018	0.077	.
	Line: Plant Species	11	2.006	0.938	0.068	0.679	
	Residuals	127	24.701		0.835		
	Total	147	29.593		1.000		
Seed 4	**Line**	7	2.416	1.774	0.081	**0.001**	***
	Plant Species	2	0.550	1.413	0.019	0.073	.
	Line: Plant Species	11	1.981	0.925	0.067	0.747	
	Residuals	127	24.713		0.833		
	Total	147	29.660		1.000		
Seed 5	**Line**	7	2.251	1.652	0.076	**0.001**	***
	Plant Species	2	0.553	1.419	0.019	0.076	.
	Line: Plant Species	11	1.980	0.925	0.067	0.765	
	Residuals	127	24.729		0.838		
	Total	147	29.513		1.000		

A–E: the different results are from the five different random seeds for rarefaction to prevent sampling bias of the ASVs. Depending on the selected ASVs, ancestral line and host plant significantly influence the microbiome. Overall, only a small amount of the total variation is explained (*R*^2^ ≈ 0.17) and most of each is explained by the ancestral spider mite line (*R*^2^ ≈ 0.08). Values in bold are considered significant as they are lower than the conventional *p*-value of 0.05. The asterisks indicate the level of significance (‘.’: *p*-value ≤ 0.10 and > 0.05, ‘*’: *p*-value ≤ 0.05 and > 0.01, ‘**’: *p*-value ≤ 0.01 and > 0.001, and ‘**’: *p*-value ≤ 0.001).

The average community dissimilarities showed the largest variation in composition across plant species for spider mite line 3, followed by lines 8 and 5 (on average 0.691, 0.634, and 0.628, respectively), whereas the lowest variation was found for spider mite line 1, followed by lines 4 and 6 (average microbiome dissimilarity of 0.548, 0.572, and 0.572, respectively). Although the average microbiome dissimilarity for the samples from the mixed line 8 was rather large, we did not see strong differences between the genetically depleted lines and the mixed lines. In general, the variation in bacterial communities associated with the spider mites living on cucumber plants was smaller (average microbiome dissimilarity of 0.598) than with those living on the other two plant species (0.609 and 0.640 for tomato and bean, respectively; Figure 5).

The Cramer-von Mises statistic revealed no consistent significant results (Supplementary Table S10) among the different rarefied datasets, indicating that none of the microbial communities were more similar to each other than could be explained by chance. Hence, the mixed lines were not more similar to a single spider mite line.

### Spider Mite Performance

**Figure 6:**
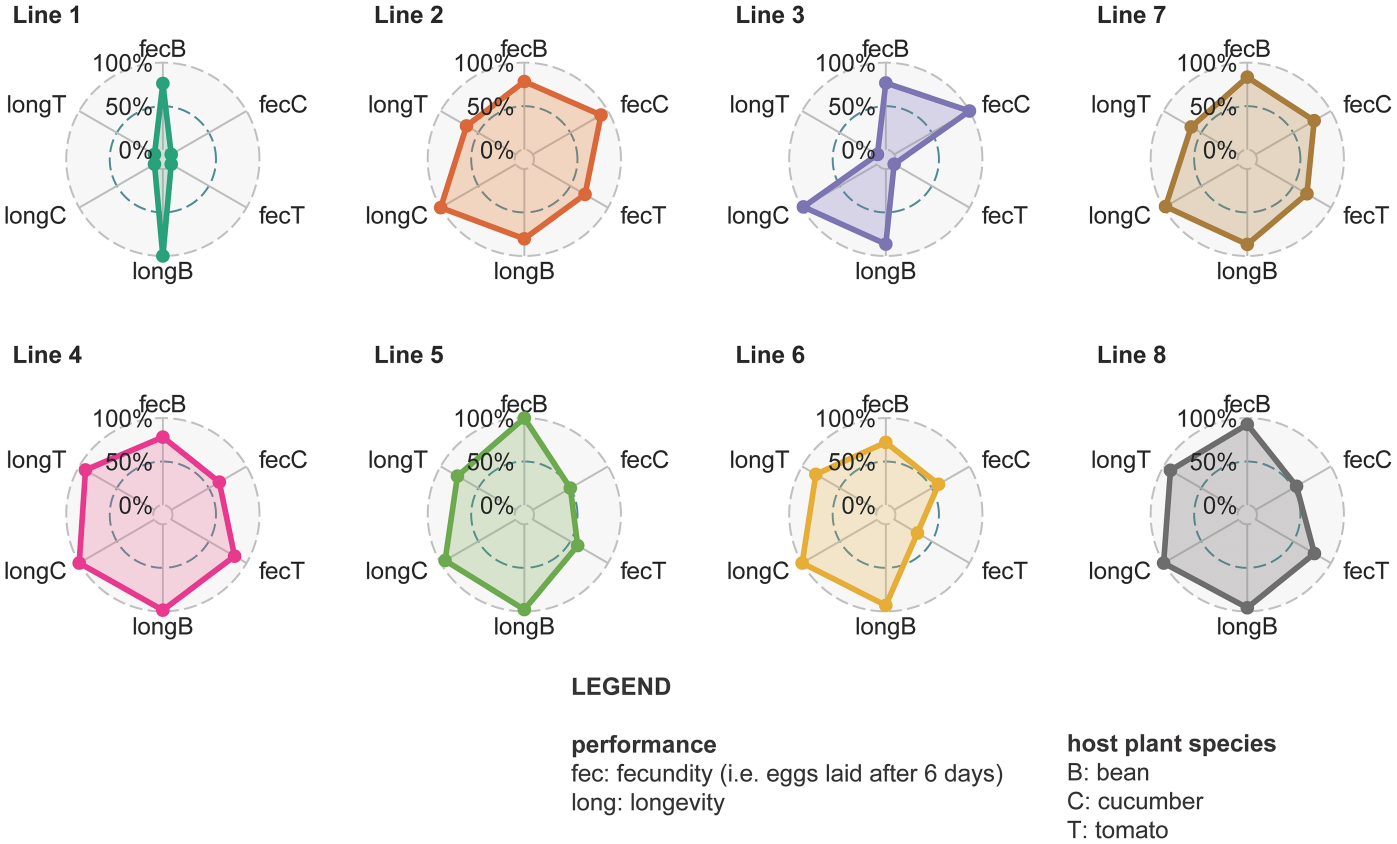
Spider plots from different lines with all measured fitness proxies (day 150). Different circles represent different spider mite lines. Line 7 is shown after line 1, 2, and 3 as it is their mixed line and the same holds for line 8 that is shown after line 4, 5, and 6. Within each circle the dots are the relative fecundity (fec) or longevity (long) on the different host plants (B is bean, C is cucumber, and T is tomato) that is scaled against the maximum value found for that trait. The larger the colored surface within the circle, the larger the adaptive potential of that line.

The fecundity assessed on bean plants was initially significantly higher than the fecundity on tomato plants for all the different spider mite lines (Figure 6; Supplementary Figures S7, S8; Supplementary Table S11). This changed at day 150 when mites living on bean and those living on tomato for lines 2, 3, and 4 showed a similar performance (Supplementary Figure S7). These particular spider mite lines thus achieved the same fecundity on tomato plants as on the ancestral bean plants. Fecundity on cucumber was initially intermediate between bean and tomato; only individuals from line 1 (*t* = 3.861 and *p* = 0.0233) had a significantly lower fecundity on cucumber than on bean plants. After 150 days lower fecundity on cucumber than on bean was observed for line 4 (*t* = 4.285 and *p* = 0.0054), line 5 (*t* = 8.133 and *p* < 0.0001), and line 6 (*t* = 3.951 and *p* = 0.0182). Different replicates from the same spider mite line obtained similar fecundity after 150 days (Supplementary Figure S8). Regarding longevity, the longest living individuals were from the populations living on cucumber, while the shortest survival probabilities were observed in the tomato populations (Figure 6; Supplementary Figure S9; Supplementary Table S12). Individuals from lines 2 and 7 had a low survival probability overall. Different replicates for the same lines showed the same trend for longevity (Supplementary Figure S8). The mixed lines did not perform better than the genetically depleted lines in terms of longevity or fecundity.

### Relation Between Performance and Microbiome Composition

All results were consistent across the different random seeds used in rarefaction, except the results where the performance matrix was created only from longevity; only for some seeds significant correlations were found on tomato. We found a strong correlation between the difference in performance of the spider mite lines and the difference in their microbiome on the two novel host plants (cucumber and tomato) based on the fecundity and longevity data combined (Table 2). This relation was not found on their initial host plant (bean) alone. A significant relation on both novel host plants was also found if only fecundity was considered. However, if only longevity was considered, the relation with the microbiome composition on cucumber plants disappeared. The significance for longevity on tomato plants depended on the ASVs that were sampled during rarefaction (i.e., the used seed).

**Table 2:** Relationship between spider mite fitness proxies and microbiome community structure, using Procrustes and Mantel tests.

	Fecundity						Longevity						Fecundity and longevity					
	Bean		Cucumber		Tomato		Bean		Cucumber		Tomato		Bean		Cucumber		Tomato	
	st.	p-v.	st.	p-v.	st.	p-v.	st.	p-v.	st.	p-v.	st.	p-v.	st.	p-v.	st.	p-v.	st.	p-v.
Euclidian	0.62	0.118	0.46	**0.006**	0.47	**0.031**	0.741	0.798	0.73	0.462	0.52	0.074	0.58	0.125	0.46	**0.006**	0.42	**0.024**
	0.61	0.080	0.45	**0.001**	0.48	**0.028**	0.721	0.686	0.70	0.196	0.46	**0.013**	0.57	0.104	0.44	**0.001**	0.41	**0.007**
	0.62	0.087	0.49	**0.003**	0.49	**0.014**	0.734	0.849	0.71	0.278	0.48	**0.021**	0.57	0.095	0.49	**0.003**	0.43	**0.001**
	0.63	0.092	0.51	**0.005**	0.48	**0.013**	0.735	0.880	0.70	0.225	0.49	**0.029**	0.58	0.102	0.51	**0.004**	0.42	**0.007**
	0.62	0.088	0.54	**0.017**	0.45	**0.004**	0.711	0.596	0.71	0.265	0.49	**0.026**	0.58	0.106	0.53	**0.017**	0.39	**0.007**
Manhattan	0.62	0.118	0.46	**0.006**	0.47	**0.031**	0.741	0.798	0.73	0.462	0.52	0.074	0.55	0.143	0.44	**0.007**	0.37	**0.017**
	0.61	0.080	0.45	**0.001**	0.48	**0.028**	0.721	0.686	0.70	0.196	0.46	**0.013**	0.54	0.115	0.42	**0.001**	0.36	**0.007**
	0.62	0.087	0.49	**0.003**	0.49	**0.014**	0.734	0.849	0.71	0.278	0.48	**0.021**	0.54	0.120	0.47	**0.003**	0.37	**0.001**
	0.63	0.092	0.51	**0.005**	0.48	**0.013**	0.735	0.880	0.70	0.225	0.49	**0.029**	0.55	0.120	0.49	**0.003**	0.36	**0.007**
	0.62	0.088	0.54	**0.017**	0.45	**0.004**	0.711	0.596	0.71	0.265	0.49	**0.026**	0.54	0.109	0.51	**0.015**	0.33	**0.007**
Euclidian	0.28	0.105	0.57	**0.014**	0.56	**0.019**	−0.133	0.758	0.03	0.462	0.31	0.146	0.27	0.106	0.57	**0.014**	0.60	**0.010**
	0.34	0.072	0.73	**0.002**	0.45	**0.026**	−0.118	0.719	0.22	0.110	0.64	**0.025**	0.34	0.081	0.73	**0.002**	0.53	**0.007**
	0.32	0.077	0.71	**0.004**	0.41	**0.018**	−0.089	0.662	0.17	0.205	0.43	0.081	0.34	0.071	0.71	**0.004**	0.45	**0.001**
	0.31	0.076	0.64	**0.008**	0.51	**0.015**	−0.185	0.849	0.24	0.114	0.49	0.058	0.30	0.084	0.64	**0.008**	0.57	**0.004**
	0.29	0.090	0.49	**0.029**	0.61	**0.008**	−0.084	0.640	0.17	0.224	0.42	0.100	0.28	0.098	0.49	**0.028**	0.66	**0.008**
Manhattan	0.28	0.105	0.57	**0.014**	0.56	**0.019**	−0.133	0.758	0.03	0.462	0.31	0.146	0.26	0.115	0.57	**0.014**	0.64	**0.010**
	0.34	0.072	0.73	**0.002**	0.45	**0.026**	−0.118	0.719	0.22	0.110	0.64	**0.025**	0.33	0.085	0.74	**0.002**	0.60	**0.004**
	0.32	0.077	0.71	**0.004**	0.41	**0.018**	−0.089	0.662	0.17	0.205	0.43	0.081	0.31	0.088	0.72	**0.003**	0.52	**0.001**
	0.31	0.076	0.64	**0.008**	0.51	**0.015**	−0.185	0.849	0.24	0.114	0.49	0.058	0.28	0.091	0.65	**0.006**	0.63	**0.004**
	0.29	0.090	0.49	**0.029**	0.61	**0.008**	−0.084	0.640	0.17	0.224	0.42	0.100	0.28	0.099	0.50	**0.026**	0.71	**0.008**

The distance matrices used for both the Procrustes and the Mantel test measure the differences between the microbiome composition of the different spider mite lines on their host plant (bean, cucumber, or tomato) and the differences between the fecundity, longevity, or both fecundity and longevity at the last time point (150 days). The significant results (*p*-value lower than 0.05) are visualized in bold. For the microbiome distance table, the unweighted UniFrac was chosen, while for the performance table two different distance measures were used (i.e., Euclidean and Manhattan). The Mantel test was done with 9,999 permutations based on the Pearson method.

On the one hand, we discovered a significant positive correlation between mean fecundity and Faith’s phylogenetic diversity (trend = 0.1370 ± 0.0485, *t* ratio = 2.826, and *p* = 0.0083) and between fecundity and species richness (trend = 0.1289 ± 0.0484, *t* ratio = 2.665, and *p* = 0.0123) on bean. On the other hand, we found a significant negative correlation on cucumber for Shannon diversity index (trend = −0.1242 ± 0.0572, *t* ratio = −2.172, and *p* = 0.0379) and species richness (trend = −0.1166 ± 0.0559, *t* ratio = −2.084, and *p* = 0.0457). No significant interactions were found between alpha diversity and longevity (Supplementary Figure S10).

## Discussion

We used experimental evolution to gain insights into the role of the microbiome in adaptation of the host to new resources. We here studied adaptation to host plants from the perspective of fitness maximization, including genetics and plasticity through interaction with microbiomes. Our results show a potential importance of the microbiome for adaptation of spider mites to novel food sources, and suggest that while both host ancestry and plant host environment may contribute to shaping the microbiome, these factors only explain a small part of the variation in the microbiome composition. In general, we did not discover large discrepancies between the mixed and genetically depleted lines. On the one hand, this might be because the genetic diversity provided in the mixed lines was still rather low for a good comparison: the genetically depleted lines were made from a population that had been maintained for many generations in the lab, meaning that the genetic diversity among spider mite lines was potentially low. On the other hand, this could be due to outbreeding depression. The genetically depleted lines were preselected to obtain the most different lines based on the bacterial part of their microbiome and genome. Mating these lines could potentially lead to heterozygote disadvantage and the break-up of coadapted gene complexes and epistatic interactions (Price andPrice and Waser, 1979; Peer and Taborsky, 2005).

### Influence of Experimental Evolution on Performance

Before the evolutionary experiment, the fecundity of the spider mites on tomato leaves was significantly lower for all genetically depleted lines compared to their fecundity on bean leaves (Supplementary Figure S7). After 150 days of evolution, some lines (lines 2, 3, and 4) managed to obtain an equal fecundity on tomato and on bean, even though all lines were genetically depleted. Interestingly, this was not found for the mixed lines; the performance on tomato was still significantly lower than on bean. We expected that mixing or outbreeding of lines would lead to an increase in fitness. However, under certain conditions, an outbreeding depression resulting in lower fecundity, is likely (Price and Waser, 1979; Peer and Taborsky, 2005). Indeed, the two-spotted spider mite is a haplodiploid species with fast exponential growth followed by population collapses, indicating higher chances for sibling mating and prolonged inbreeding. It is therefore probable that the species is better protected against inbreeding depression than outbreeding depression (e.g., *via* purifying selection on recessive deleterious alleles). In fact, a study of Tien et al. (2015) found no negative effect of inbreeding on oviposition rate in *T. urticae* anymore at an inbreeding coefficient, *F*, of 0.5 (i.e., selfing).

Adaptation of *T. urticae*, which was maintained on a single host plant for many generations in the lab, to tomato plants has been observed in previous research as well (Magalhães et al., 2007; Alzate et al., 2017). We expected tomato plants to be challenging host plants due to their induced responses and glandular trichomes (Lucini et al., 2015; Godinho et al., 2016). The differences among lines was not surprising as intraspecific variation within *T. urticae* has been found to result in different responses to plant defenses; some lines may induce defenses to which they are susceptible, others induce but are not susceptible, while certain lines may suppress defenses (Kant et al., 2008). The fecundity on cucumber plants was initially intermediate between the fecundity on bean and tomato, but not significantly different from the bean leaves. Only line 1 had a significant lower fecundity on both novel host plants and did not survive the evolutionary experiment on the novel host plants.

Longevity was highest on cucumber plants, followed by bean and tomato (Fig. S9). The higher mortality on the hostile tomato plants was expected, but longer lifetimes on cucumber were unanticipated. Also, no interaction effects between spider mite line and host plant species were found, which indicates that certain spider mite lines survived longer but independently of their host plant species.

### Effect of Host Ancestry on the Bacterial Communities

The bacterial communities associated with our focal species are partly horizontally transmitted from the plant environment, but also inherited from parents to offspring, given the significant effect of the spider mite line on microbiome composition in the PERMANOVA. Heritability of microbiomes ranges from entirely faithful as seen in intracellular infection of oocytes to completely unfaithful in for instance marine sponges (Bruijning et al., 2022). This flexibility could be beneficial to create variable microbiomes for rapidly changing environments or for different life stages (Henry et al., 2021; Bruijning et al., 2022).

Many microorganisms, such as *Wolbachia* spp. and *Rickettsia* spp., are intracellularly transmitted in the egg cytoplasm (Hong et al., 2002; Veneti et al., 2005; Zilber-Rosenberg and Rosenberg, 2008). We found a high abundance of these commonly found bacteria in our populations of *T. urticae*, which could explain the importance of host ancestry to microbiome composition. It is, however, possible that also other ASVs were vertically transmitted, particularly because the distance metric we used (unweighted UniFrac) does not take abundance into account and only on average 7.7% of all ASVs belonged to the Rickettsiales family. While spider mites reproduce by laying eggs, it is unknown whether the surface of the eggs is a transgenerational carrier of bacteria. This is known for some, yet unsupported for other species (Romero and Navarrete, 2006; van Veelen et al., 2018).

Although the contribution of host ancestry to microbiome composition was significant, it was very small, which may be due to the fact that individual lines were created from the same population. The used mite strains already persisted for more than 40 generations and had been feeding on the same host plant species for more than 400 generations. Such co-feeding does not necessarily lead to transfection or homogenization of the microbiome (Oliver et al., 2010). The (albeit small) contribution of ancestry indicates that certain microorganisms may have been vertically transmitted, but another option is a strong filter of the genetically depleted spider mite line creating microbial differences between individuals.

We aimed to provide results from the initial bacterial communities of the different lines, but only have data from four spider mite lines due to low population sizes and failed DNA extractions. On the one hand, two spider mite lines (lines 1 and 6) seem to be less affected by the host plant species or the measured time point (Supplementary Figure S2), which could indicate a more stable bacterial community. On the other hand, the other two lines show a small shift due to host plant species (line 2) or both time and host plant species (line 4; Supplementary Figure S2). Interestingly, these latter two lines obtained a similar fecundity on tomato compared to their initial host plant after the experimental evolution, while this did not happen in lines 1 and 6 (Supplementary Figure S7).

### Effect of Host Environment (Host Plant Species) on the Bacterial Communities

The standardized number of ASVs found in bean plants was higher than the ASVs in the novel host plants, cucumber and tomato (Figure 3). Especially the small number of shared ASVs may indicate a strong environmental filter for each host plant (Diamond, 1975; Takeuchi et al., 2015). Besides, an indirect influence of the host plant species on the microbiome variation is likely due to differences in spider mite population sizes and inbreeding. Indeed, lower numbers of ASVs on tomato plants could be linked to the smaller population sizes on these host plant species.

In our study, the plant species plays a minor role in shaping the microbiome: as, on average, only 2% of the total variation was explained by host plant species in the PERMANOVA. Acquiring bacteria through the environment from other members in the community is known to occur frequently (Oliver et al., 2010; Henry et al., 2013) and is a potential advantage for adaptation. In contrast to genetic adaptation, acquiring microorganisms might even happen throughout an individual’s life (Zilber-Rosenberg and Rosenberg, 2008). An example of such horizontal transmission can be found in aphids, where horizontally transmission occurred by feeding on a diet including symbionts, potentially aided by the sugar-rich liquid or honeydew (Darby and Douglas, 2003). A similar horizontal transmission is possible in spider mites through foraging close to fecal pellets or co-feeding from plant tissues (Chrostek et al., 2017). An example of this plant-mediated transfer has for instance been found for *Cardinium* in aphids (Gonella et al., 2015).

### Bacterial Communities Correlate With Spider Mite Performance

The difference in bacterial diversity between lines was clearly associated with the difference in mite fecundity and their overall performance (fecundity and longevity) on the two novel host plants. Fecundity and alpha bacterial diversity also showed to be related. We therefore dare to suggest that the microbial community is not just transient, but that it could play a role in the life history traits of the host. More importantly, as there was no correlation between the genetic distances and differences in performance between spider mite lines (Supplementary Table S13), this result may be independent of the genetic background of the mites. Alternatively, it is also possible that both the microbiome and the performance of the mites respond to parts of the host genome that were not screened with the microsatellites. More research is necessary to further unravel the influence of the genetic background and microbiome on spider mite adaptation. Despite the fact that we obtained similar results for fecundity and longevity for the different replicates within the same spider mite line (see Supplementary Figure S8), we cannot rule out drift effects. However, drift may lead to evolution, but rarely to adaptation. We therefore believe that the correlation between the microbial composition and performance is not the result of drift.

The large abundance of Rickettsiales in our samples may partly explain the relation between performance and the microbiome composition. Endosymbionts such as *Wolbachia* (belonging to the Rickettsiales), *Cardinium*, and *Spiroplasma* have been found to inconsistently alter mite performance and plant resistance (Staudacher et al., 2017). Also, *Wolbachia* might help or hinder mite performance which strongly depends on the host plant (Zélé et al., 2018b). Interestingly, *Wolbachia* has been observed to be most prevalent on bean and to hamper performance on Solanaceous plants (Zélé et al., 2018b). This could explain why we found such high *Wolbachia* abundances in our populations, which had been maintained on bean for more than 400 generations, and also why two spider mite lines failed to survive on the tomato plants. To further determine the role of the Rickettsiales, we redid the analysis including only the reads from this specific order. We found no consistent correlation between performance and this subset of the bacterial composition (Supplementary Table S14), which shows the potential importance of the other orders in the microbiome. Identifying which specific genera or species play a role in spider mite performance on novel host plants will be a fascinating area of exploration, and future studies should investigate this research topic.

Besides Rickettsiales, Enterobacteriales might also affect the performance of the host. This was the fourth most abundant order in our samples. In aphids some bacteria from this order seem to be important for defense against fungi (e.g., *Regiella insecticola*) or parasitoids (e.g., *Hamiltonella defensa*; Oliver et al., 2005, 2010; Scarborough et al., 2005; Łukasik et al., 2013; Weldon et al., 2013; Dykstra et al., 2014; Oliver and Higashi, 2019). Facultative symbionts are known to both interfere with and promote reproduction, and boost survival (Oliver et al., 2010). For instance, some *Wolbachia* strains have been reported to decrease fecundity in *T. urticae* (Vala et al., 2003), but in the same study no influence on longevity was found. However, we did find some influence of the microbiome on longevity of *T. urticae*, what leads to three potential alternatives for this discrepancy: (i) *Wolbachia* may not be causing the described effect, (ii) the influence of the host plant species as we mainly saw an effect on tomato plants and the former study was conducted on cucumber plants, or (iii) the tetracycline and heat treatments used (Vala et al., 2003) for clearing the mites of *Wolbachia* infection might have partly affected other components of the microbiome.

It is interesting that we find relationships between difference in performance (fecundity and longevity) between lines and difference in microbiome composition on the novel host plants, but not on the ancestral plant species. Because these spider mite populations have been reared on bean plants for over 400 generations, the most drastic improvements to their fitness and performance on bean plants have likely already occurred. This can explain why no relationship between performance and microbial composition was found on bean plants. Also, the amount of variation in performance found between the lines on bean plants was rather minor compared to the differences in fecundity and longevity found on the other host plants, which makes it difficult to reveal clear signals. Although the overall variation in the bacterial community was larger in bean compared to the novel host plants (as seen in the average microbiome dissimilarities), slight differences in the microbiome may have been beneficial for the adaptation to novel host plants. This suggests that the microbiome could be mainly of importance when adapting to a new environment. Adaptation through the microbiome could potentially provide a rapid response to novel environments when genetic adaptation would be too slow, similarly to how phenotypic plasticity can aid adaptation through “plastic rescue” (Snell-Rood et al., 2018; Fox et al., 2019).

The microbial variation is only partly explained by host genetics and diet, but is to a large extent influenced by other unknown factors. Environmental factors such as temperature and altitude are for instance known to be crucial for the prevalence of endosymbionts in *Tetranychus* spp. (tested in *T. truncates*, Zhu et al., 2018), to standardize many factors, we performed our experiments under the same climate-controlled conditions. However, we cannot control for random processes such as drift that may influence assembly processes (Vellend, 2010).

We decreased potential biases in contaminants from host plant species (e.g., phyllosphere microbial communities) by only comparing bacterial communities of spider mite lines reared on the same host plant species. We neither compared the fecundity and longevity of spider mite populations reared on novel host plant species with their performance on the ancestral host plant species. Such a comparison would likely lead to an overestimation of the performance on the novel host plant species, because juvenile and maternal effects could not be standardized in our experiments (i.e., we did not have a two generations common garden given that this would indicate many bacterial generations). Moreover, maternal effects may be stronger in certain populations compared to others which would complicate the interpretation of the results. Hence, we only compare spider mite lines on the same host plants at the same time points.

In conclusion, although the evolutionary interests of the microorganisms might not be entirely in line with those from their host, our results suggest that the microbiome could play a role in the performance of spider mites on novel host plants. The potential effect of the microbiome on its host phenotype linked with the significant influence of host genetics on the bacterial community composition, implies a possible advantage of including microbiome data in heritability studies (Douglas et al., 2020). Furthermore, we found that the composition of the spider mite bacterial communities depends partly on host genetics and on the host’s environment (i.e., the plant it feeds on), but mostly on other, yet unknown, factors. We speculate that founder and priority effects may be important, where the specific bacterial species or even the order of uptake of the bacterial community members influences the final community (Fukami, 2015; Debray et al., 2022); future studies using axenic lines could provide insights. Multiple bacteria could perform similar functions or work together forming functional groups or guilds, hence, guild-based analyses could be meaningful (Wu et al., 2021). Another possibility is the existence of a core group while the other members of the bacterial communities are the result of stochastic or neutral sampling processes. As our study is only correlative, we do not want to take strong conclusions, but advocate that the microbiome should be considered when studying adaptation. Adaptation through the microbiome could be a fast solution under rapidly changing conditions (Snell-Rood et al., 2018).

## Data Availability Statement

The datasets presented in this study can be found in online repositories. The names of the repository/repositories and accession number(s) can be found at: http://www.ncbi.nlm.nih.gov/bioproject/596721, NCBI GenBank Sequence Read Archive (SRA) database as project PRJNA596721. The script and data for the manuscript are available on DataVerseNL (https://doi.org/10.34894/AMTPZS).

## Author Contributions

All authors contributed to the idea of the experiment, discussions, and revisions. KB and CM did the pilot study. KB, HK, and TE performed the final experiments. KB and HK did the statistical analysis. KB wrote the draft of the manuscript. All authors contributed to the article and approved the submitted version.

## Funding

RE and KB thank the Netherlands Organization for Scientific Research (NWO) for financial support through a VICI grant (VICI grant number 865.13.00) and an NWA-ORC grant (grant number 400.17.606/4175). KB thanks the Special Research Fund (BOF) of Ghent University and the Ubbo Emmius sandwich program of the University of Groningen. KB, DB, and RE thank the FWO for the obtained funding from the research community “An eco-evolutionary network of biotic interactions,” project G018017N and junior FWO fellowship 12T5622N. TE thanks the Erasmus Mundus Master Programme in Evolutionary Biology (MEME) for the opportunities and funding provided.

## Conflict of Interest

The authors declare that the research was conducted in the absence of any commercial or financial relationships that could be construed as a potential conflict of interest.

## Publisher’s Note

All claims expressed in this article are solely those of the authors and do not necessarily represent those of their affiliated organizations, or those of the publisher, the editors and the reviewers. Any product that may be evaluated in this article, or claim that may be made by its manufacturer, is not guaranteed or endorsed by the publisher.
