# Supplementary material for: Microbiome Heritability and Its Role in Adaptation of Hosts to Novel Resources
Source: Front Microbiol. 2022 Jul 5;13:703183. doi: 10.3389/fmicb.2022.703183 (PMC9296072; doi:10.3389/fmicb.2022.703183)
Supplement: Supplementary file 1 [file Data_Sheet_1.PDF]

SUPPLEMENTARY DATA

Microbiome heritability and its role in adaptation of hosts to novel resources

Karen Bisschop†, Hylke H. Kortenbosch†, Timo J. B. van Eldijk, Cyrus A. Mallon, Joana F. Salles, Dries Bonte, and Rampal S. Etienne

MATERIALS AND METHODS SUPPLEMENTARY 2

Materials and Methods S1: Selecting the six most divers genetically depleted lines 2

Microsatellites 2

Denaturing Gradient Gel Electrophoresis (DGGE) 3

FIGURES 7

Figure S1: Number of replicates for performance tests: (a) at day 0 and (b) at day 150. 7

Figure S2: PCoA plots to compare bacterial communities at start and final sampling point. 8

Figure S3: Rarefaction curves. 9

Figure S4: Comparison of alpha diversity. 10

Figure S5: Overview of the most abundant orders per spider mite line and plant species. 11

Figure S6: PCoA plots based on the unweighted UniFrac, colored by plant species. 12

Figure S7: Fecundity of spider mites for the different plant species and spider mite lines after (a) 0 and (b) 150 days. 13

Figure S8: Fecundity and longevity for the different spider mite lines and replicates at day 150. 14

Figure S9: Survival probability of the spider mites for the different (A) spider mite lines and (B) plant species at day 150. 15

Figure S10: Correlations between alpha diversity metrics and fecundity/longevity (day 150). 16

TABLES 17

Table S1: Overview of the total number of microbiome samples in the rarefied datasets. 17

Table S2: Relationship between spider mite fitness proxies and microbiome community structure, using Procrustes and Mantel tests. 18

Table S3: Entire overview of all ASVs per plant and spider mite line (TableS3.xlsx) 19

Table S4: Model selection for phylogenetic distance. 20

Table S5: Model summary and significant pairwise comparisons for phylogenetic distance. 21

Table S6: Model selection for Shannon diversity. 22

Table S7: Model summary and significant pairwise comparisons for Shannon diversity. 23

Table S8: Model selection for species richness. 24

Table S9: Model summary and significant pairwise comparisons for species richness. 25

Table S10: Output libshuff method in Mothur, using the Cramer-von Mises test statistic. 26

Table S11: Model selection for fecundity (day 150) on the different plant species and for the different spider mite lines. 29

Table S12: Model for longevity (day 150). 30

Table S13: Relationship between spider mite fitness proxies and genetic background based on microsatellites, using Procrustes and Mantel tests. 31

Table S14: Relationship between spider mite fitness proxies and microbiome community structure (only including the Rickettsiales order), using Procrustes and Mantel tests. 32

MATERIALS AND METHODS SUPPLEMENTARY

Materials and Methods S1: Selecting the six most divers genetically depleted lines

Microsatellites

We aimed to select the six most genetically diverse lines out of nine spider mite lines (i.e., more diversity among lines, not within the genetically depleted lines). Therefore, we extracted the DNA of ten adult female per spider mite line by adding 10 µL of QuickExtractTM DNA Solution 1.0 and 1 µL proteinase K to a PCR strip with one adult female per well. After homogenization of the solution, another 10 µL QuickExtractTM was added. This solution was heated overnight at 50-55°C and followed by a final heating at 98°C for 2 minutes.

Afterwards a pre-PCR was performed. We used 1 µL of the DNA extract, with 2 µL MultiPlex (Qiagen® Multiplex PCR kit cat. No. 206143), and 2 µL of PrimerMix. Two different primer mixes were used for a total of ten microsatellite loci diluted in MQ (TuCT09, TuCT37, TuCT67, Tu27, TuCT18, TuCT73, TuCA83, Tu35b, TuCT17, and Tu16; primer sequences from Navajas and colleagues (2002) and Uesugi and Osakabe (2007). The PCR program was 95°C for 15 minutes; 35 cycles of a denaturing phase (94°C for 30 sec), annealing phase (57°C for 90 sec), and extending phase (72°C for 60 sec); and 60°C for 30 minutes.

The sequencing was performed by Andy Vierstraete (Ghent University) and we analyzed the obtained FASTA files with Geneious. The different allele frequencies per spider mite line are presented in Table i.

Table i: Allele frequencies by populations. 'Line' indicates the different genetically depleted spider mite lines used in this study. The labels of the initial nine lines that were tested are given below the line numbers (e.g. U53). The alleles that were found in only one spider mite line are indicated in green. Line U31 has been contaminated as more than two alleles were found for Tu35b (in red). Line U33 failed for all but one loci. The primer sequences from the ten microsatellite loci are obtained from Navajas et al., (2002) and Uesugi and Osakabe (2007).

	Line→	3	6	2	5				1	4
↓Loci	↓Alleles	U53	U51	U44	U42	U34	U33	U31	U24	U13
TuCT09	97	0.100	1.000	1.000	1.000	0.000	0.000	0.143	0.350	1.000
	116	0.900	0.000	0.000	0.000	1.000	0.000	0.000	0.000	0.000
	127	0.000	0.000	0.000	0.000	0.000	0.000	0.857	0.650	0.000
TuCT37	118	0.000	0.550	0.400	0.000	0.000	0.000	0.125	0.000	0.000
	124	1.000	0.450	0.600	1.000	1.000	0.000	0.875	0.300	1.000
	126	0.000	0.000	0.000	0.000	0.000	0.000	0.000	0.700	0.000
TuCT67	84	0.000	0.700	0.000	0.000	0.000	0.000	0.000	0.000	0.000
	88	0.200	0.000	0.000	0.167	0.000	0.000	0.000	0.000	0.250
	90	0.800	0.300	1.000	0.000	1.000	0.000	0.875	0.000	0.000

	94	0.000	0.000	0.000	0.833	0.000	0.000	0.000	0.600	0.000
	96	0.000	0.000	0.000	0.000	0.000	0.000	0.000	0.000	0.750
	98	0.000	0.000	0.000	0.000	0.000	0.000	0.125	0.400	0.000
Tu27	66	0.400	1.000	0.650	0.722	1.000	1.000	0.688	1.000	0.700
	69	0.600	0.000	0.350	0.278	0.000	0.000	0.313	0.000	0.300
TuCT18	289	0.643	0.375	0.000	0.750	0.000	0.000	0.550	0.000	0.000
	293	0.357	0.625	0.556	0.250	0.000	0.000	0.250	1.000	0.438
	297	0.000	0.000	0.000	0.000	0.000	0.000	0.000	0.000	0.563
	301	0.000	0.000	0.444	0.000	0.000	0.000	0.200	0.000	0.000
TuCT73	106	0.000	0.000	0.000	0.000	0.000	0.000	0.000	0.000	0.278
	108	1.000	1.000	1.000	1.000	1.000	0.000	1.000	1.000	0.722
TuCA83	202	0.000	0.000	0.000	0.000	0.000	0.000	0.450	0.000	0.000
	204	1.000	1.000	0.611	0.444	1.000	0.000	0.550	0.550	1.000
	206	0.000	0.000	0.389	0.556	0.000	0.000	0.000	0.450	0.000
Tu35b	104	0.000	0.556	0.000	0.000	0.000	0.000	0.100	0.000	0.556
	107	1.000	0.444	1.000	1.000	1.000	0.000	0.400	1.000	0.444
	110	0.000	0.000	0.000	0.000	0.000	0.000	0.500	0.000	0.000
TuCT17	293	0.375	0.000	0.000	0.000	0.500	0.000	0.750	0.000	0.000
	297	0.625	1.000	1.000	1.000	0.500	0.000	0.250	0.300	0.667
	301	0.000	0.000	0.000	0.000	0.000	0.000	0.000	0.700	0.000
	311	0.000	0.000	0.000	0.000	0.000	0.000	0.000	0.000	0.333
Tu16	141	0.438	1.000	0.000	0.333	0.000	0.000	1.000	1.000	0.000
	147	0.563	0.000	0.000	0.667	0.000	0.000	0.000	0.000	1.000

In total, we found 32 different alleles for the ten tested loci. In one of the genetically depleted lines (U31, **Table i**), we found three different alleles for Tu35b, which could be caused by contamination. We did therefore not consider this line. This lead to a total of 30 different alleles with three lines containing unique loci; U13, U24, and U53.

The two batches we selected were U24, U44, and U53 for batch one (spider mite lines 1-3 containing 24 of the 30 different alleles) and U13, U42, and U51 for batch two (spider mite lines 4-6 containing 23 out of 30 different alleles).

<u>Denaturing Gradient Gel Electrophoresis (DGGE)</u>

Besides selecting genetically diverse spider mite lines, we also aimed to select diverse lines based on their microbial composition. Therefore, we performed a PCR-DGGE analysis targeting the 16S rRNA.

DNA of the spider mites was extracted as explained in the manuscript. We performed quantification of DNA with Quant-iT™ PicoGreen ® from Invitrogen. In total 2 ng of DNA was used for the preceding PCR to amplify the 16S rRNA gene (**Table ii;** primers from Brons & Van Elsas, 2008). The steepness of the gradient of denaturants (mixture of urea and

formamide) for the DGGE ranged from 45% to 60%. The results were analyzed with Gelcompar (visualization in **Fig. i**).

Table ii: PCR protocol targeting 16S rRNA for DGGE. The initial DNA used in the reaction was 2 ng.

		1 reaction
H2O		0 µL
Buffer	10x	5 µL
MgCl2	25 mM	7,5 µL
Formacide	1	0,5 µL
dNTPs	10 mM	4 µL
F968-GC	10 µM	4 µL
R1401.1b	10 µM	4 µL
Taq	5 U/ul	1 µL
DNA		24 µL

Program		
95 °C	5 min	
94 °C	1 min	decreased by 1°C/cycle (10 cycles)
60 °C	1 min	
72 °C	2 min	
94 °C	1 min	20 cycles
55 °C	1 min	
72 °C	1 min	
72 °C	10 min	

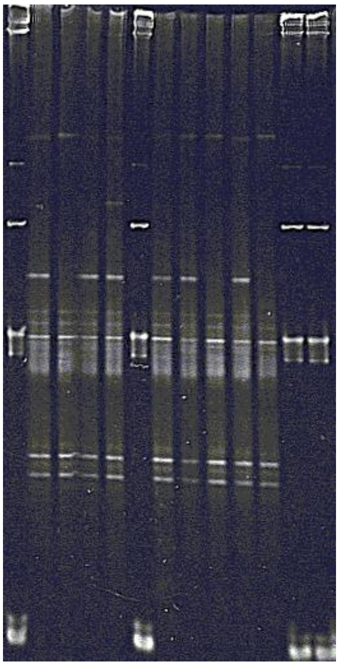

1: marker
2: U24
3: U51
4: U13
5: U33
6: marker
7: U42
8: U31
9: U44
10: U53
11: U34
12: marker
13: marker

Figure i: results DGGE to detect lines with different microbial communities. Lane 1, 6, 12, and 13 are the marker as indicated on the right.

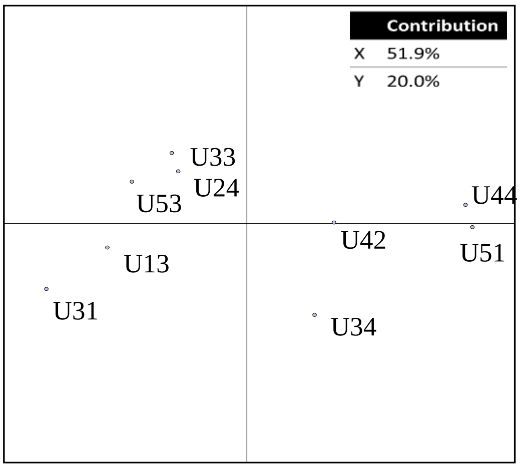

Figure ii: PCoA plot based from the DGGE (16S rRNA gene) using Gelcompar.

References

Brons, J.K. & Dirk Van Elsas, J. 2008. Analysis of bacterial communities in soil by use of denaturing gradient gel electrophoresis and clone libraries, as influenced by different reverse primers. *Appl. Environ. Microbiol.* **74**: 2717–2727.

Navajas, M., Perrot-Minnot, M.J., Lagnel, J., Migeon, A., Bourse, T. & Cornuet, J.M. 2002. Genetic structure of a greenhouse population of the spider mite Tetranychus urticae: spatio-temporal analysis with microsatellite markers. *Insect Mol. Biol.* **11**: 157–165. John Wiley & Sons, Ltd (10.1111).

Uesugi, R. & Osakabe, M.H. 2007. Isolation and characterization of microsatellite loci in the two-spotted spider mite, Tetranychus urticae (Acari: Tetranychidae). *Mol. Ecol. Notes* **7**: 290–292. John Wiley & Sons, Ltd (10.1111).

Materials and Methods S2: PCR protocol and primer sequences

PCR protocol. The upper table provides the necessary reactants and their respective volumes per PCR reactions. The lower table gives information about the used PCR program.

Reactants	µl
dNTP mix (10 mM)	0.5
Buffer 3 (Roche)	2.5
Forward Primer (10µM)	0.65
Reverse Primer (10µM)	0.65
MgCl2 (25 mM)	1.7
BSA (20mg/ml)	0.25
T4 gene protein (5 mg/ml)	0.05
Formamide	0.25
Taq Roche	0.15
Total Mastermix	6.7
DNA solution (total of 2.5 ng)	18.3
Total per PCR reaction	25.0

94°C – 1 minute	
94°C – 1 minute (denaturing phase)	35 cycles
58°C – 1 minute (annealing phase)	
72°C – 1 minute (extending phase)	
72°C – 10 minute	
20°C – till end	

Illumina adapters and primer sequence targeting the V3-V4 region of the 16S rRNA gene. The Illumina adapter is underlined, while the primer sequence is given in bold.

V3 – V4 16S rRNA: Full primer sequence

Forward primer 5’ CTTTCCCTACACGACGCTCTTCCGATCT**ACGGRAGGCAGCAG**

Reverse primer 5’ GGAGTTCAGACGTGTGCTCTTCCGATCT**TACCAGGGTATCTAATCCT**

FIGURES

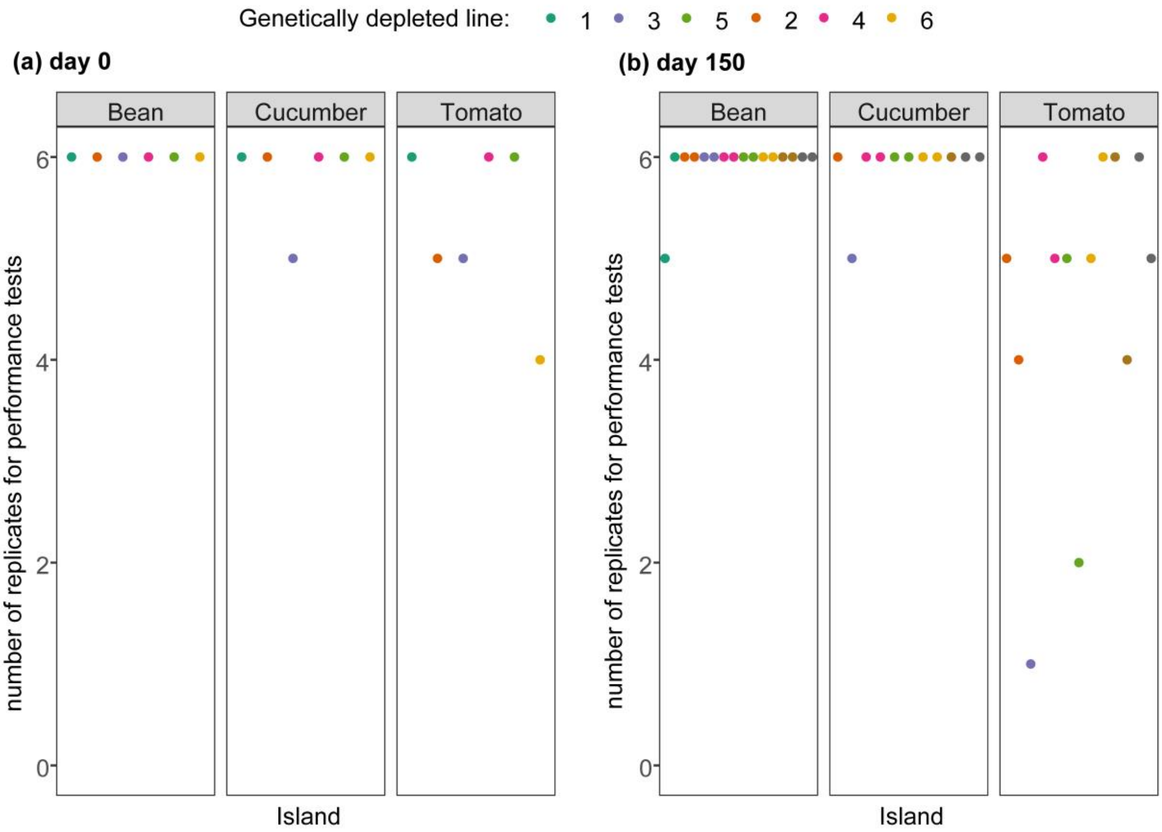

Figure S1: Number of replicates for performance tests: (a) at day 0 and (b) at day 150. The different islands are along the *x*-axis, while the *y*-axis represents the number of individual females that could be tested for fecundity/longevity tests. The colours visualize the different genetically depleted lines. Each panel gives the result per host plant species. In (a) all females were taken from bean to test on the three different plant species, in (b) the females were taken from their respective islands.

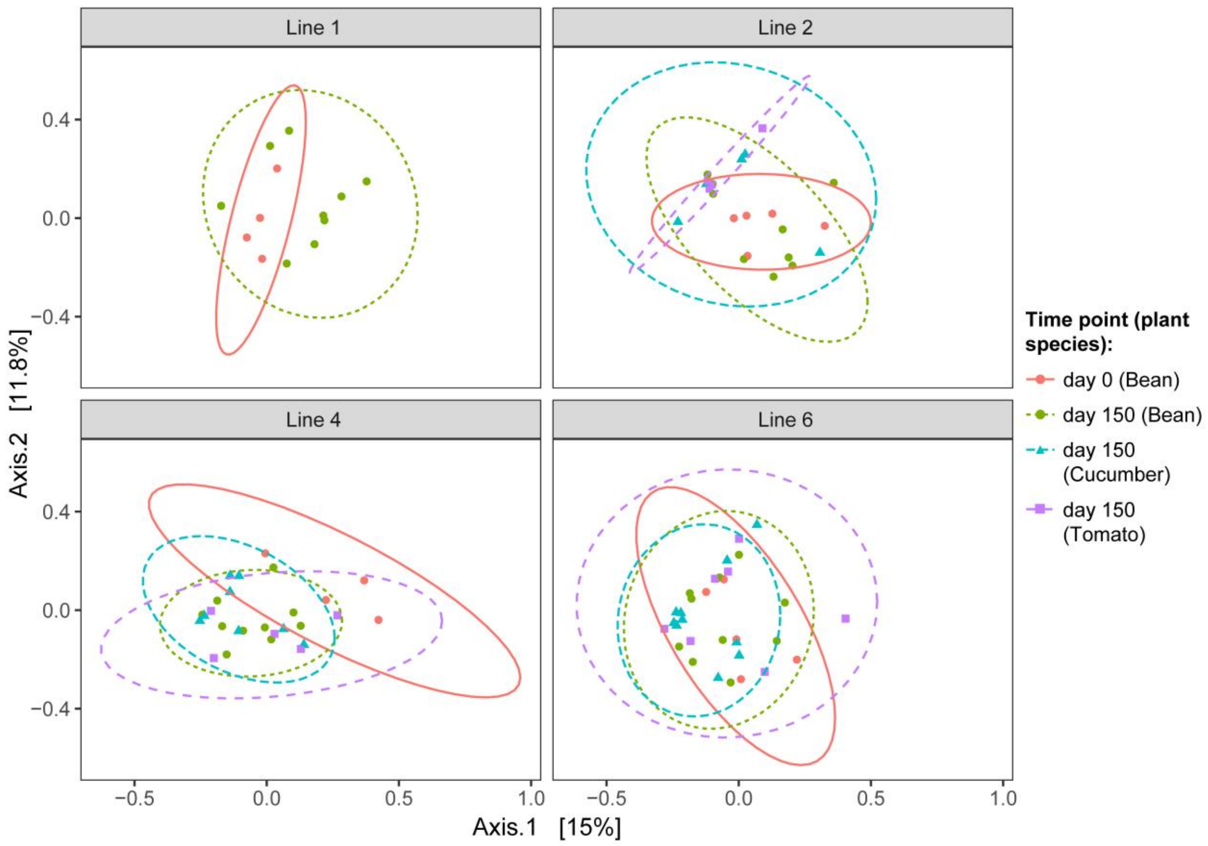

Figure S2: PCoA plots to compare bacterial communities at start and final sampling point.

The results for genetically depleted lines 1, 2, 4, and 6 are shown in facets. Lines 3 and 5 are missing due to failed DNA extractions or low numbers of sequencing reads. Each point represents a sample, the shapes indicate the different plant species (circles: bean plants, triangles: cucumber plants, squares: tomato plants) and the interaction between colors and shapes specify the different time points and plant species (red circles: bean day 0, green circles: bean day 150, blue triangles: cucumber day 150, and purple squares: tomato day 150). The ellipses show the 95% confidence level under a multivariate t-distribution.

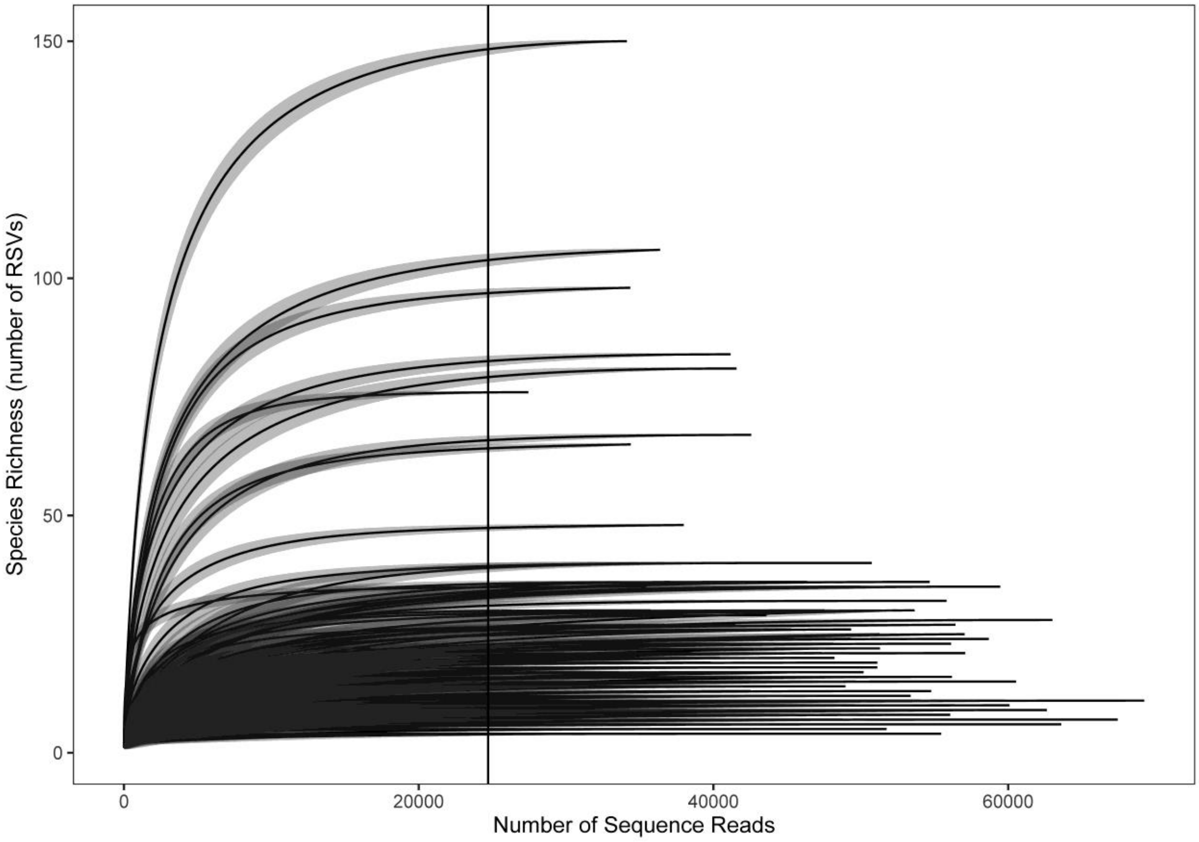

Figure S3: Rarefaction curves.

The species richness plotted against the number of reads per sample. This plot was used to choose the sequence depth for rarefaction (depth was 24712 reads indicated with the vertical line).

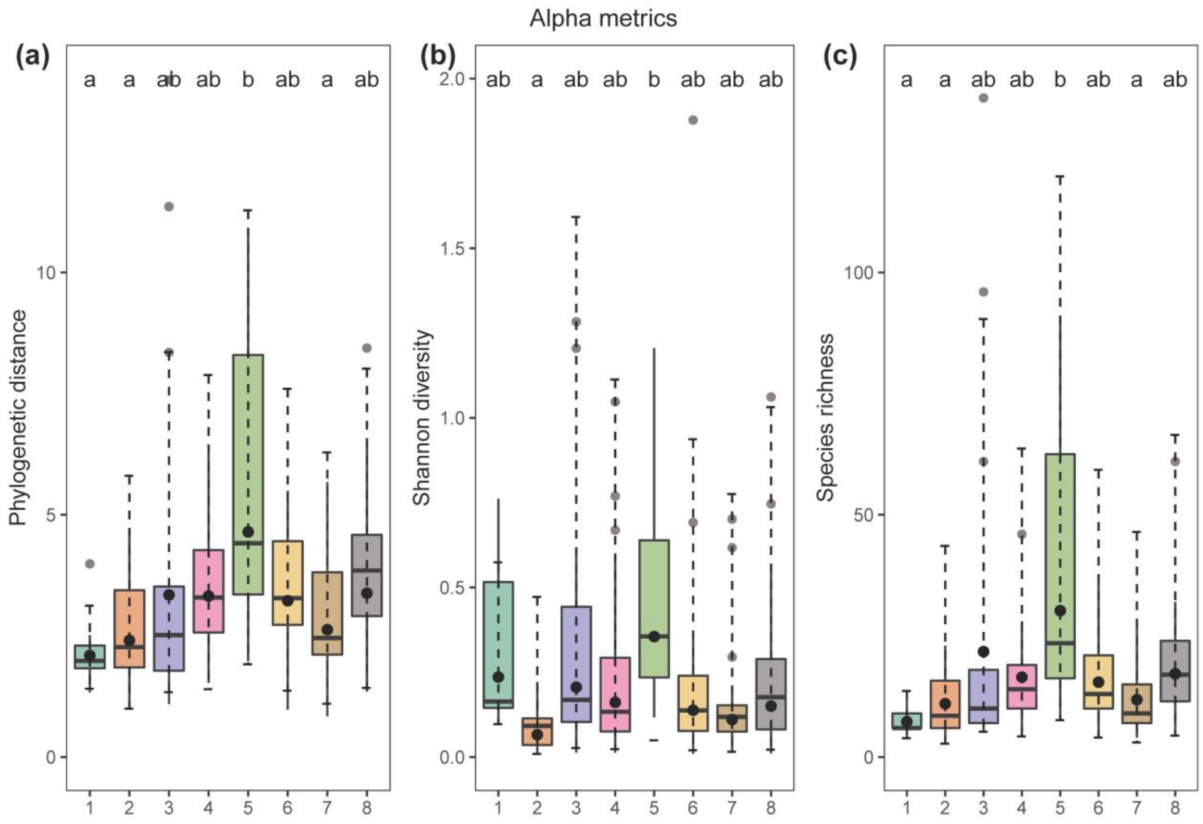

Figure S4: Comparison of alpha diversity.

The alpha diversity calculated through three different metrics: (a) Phylogenetic distance, (b) Shannon diversity, and (c) species richness. The different spider mite lines are visualized on the x-axis. The plant species were not included in the most parsimonious model for the three metrics, hence, the plotted results are for all plant species. Significant differences among lines are represented by letters. The boxplots represent the raw data, while the black dots and dashed lines are the mean and standard deviation of the model output. Overall, spider mite line 5 has a relative higher alpha diversity, while spider mite lines 1, 2, and 7 have a lower alpha diversity.

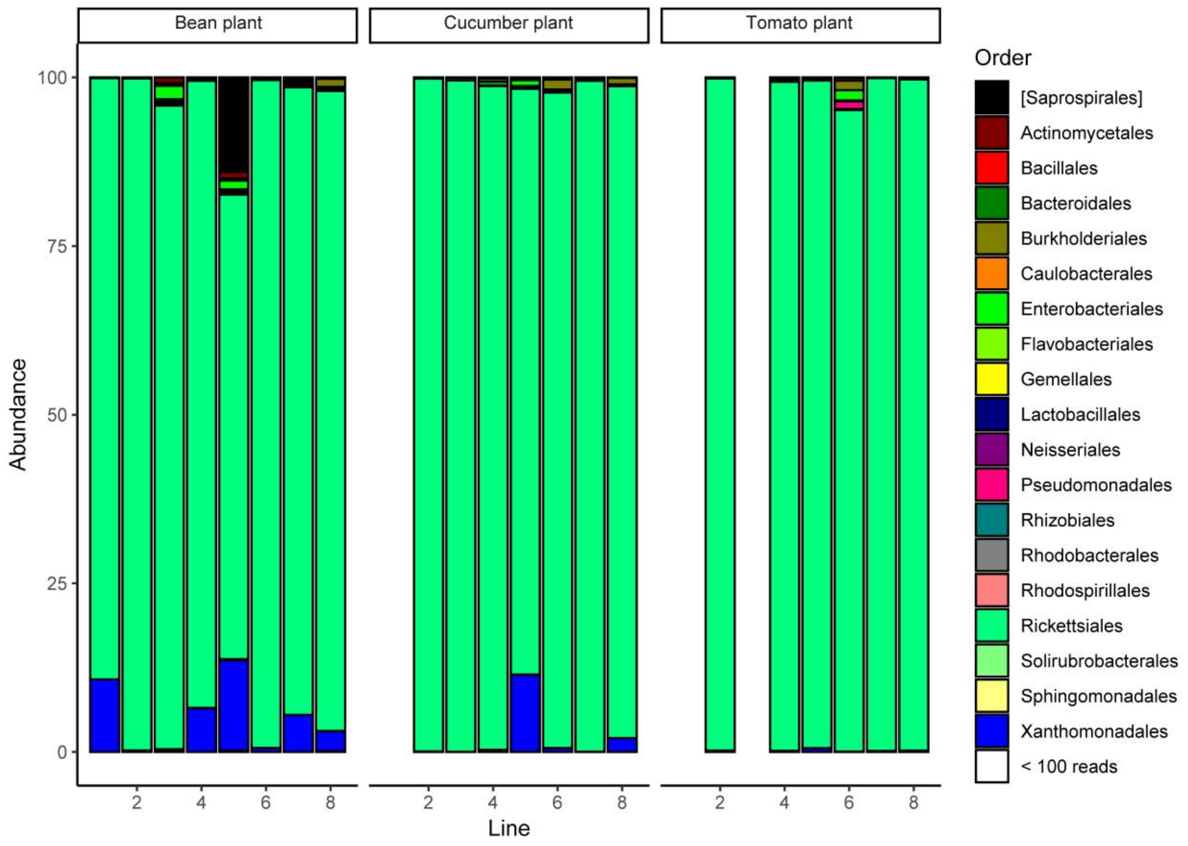

Figure S5: Overview of the most abundant orders per spider mite line and plant species. All orders are presented from ASVs with more than 100 reads in the total rarefied dataset. The most abundant taxonomic order is clearly the Rickettsiales, followed by the Xanthomonadales, the Saprospirales, the Enterobacteriales, and the Burkholderiales. No difference in most abundant orders was found between different seeds.

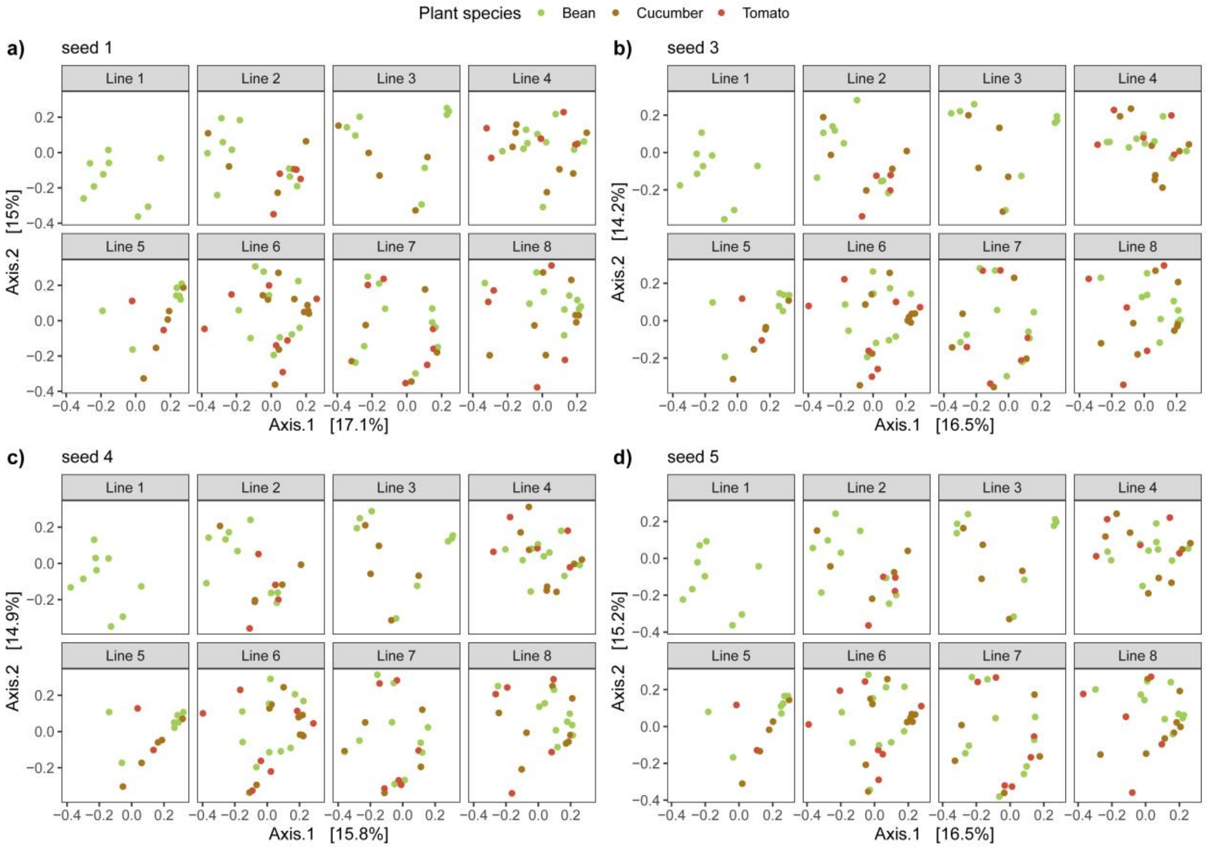

Figure S6: PCoA plots based on the unweighted UniFrac, colored by plant species.

The figure consists of four different plots showing the output for the rarefied datasets under different seeds (a-d). The eight panels within the plots represent the different spider mite lines under the same ordination. The PERMANOVA showed that only little of the variation is explained by the ancestral line and the host plant. The result for seed 2 is shown in Fig. 4.

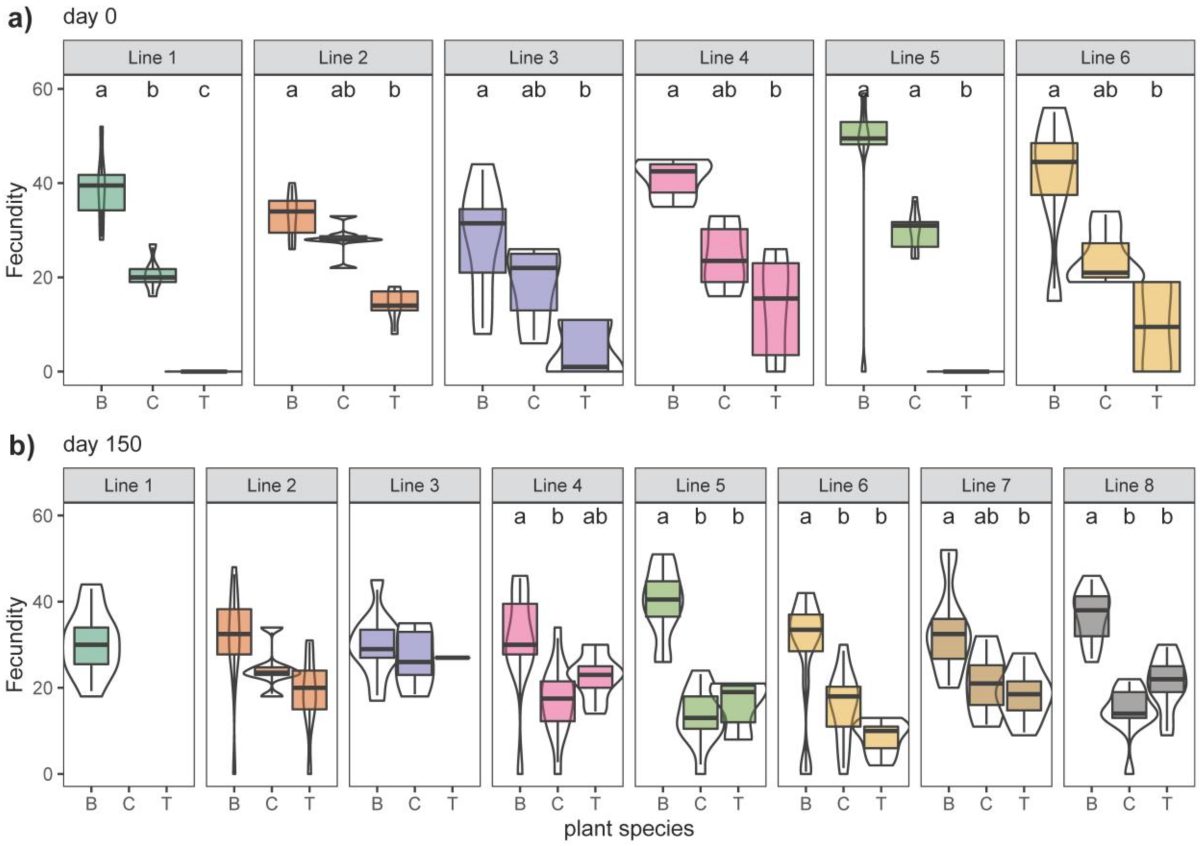

Figure S7: Fecundity of spider mites for the different plant species and spider mite lines after (a) 0 and (b) 150 days.

The violin and boxplots represent the fecundity assessments, the quantiles are set at 0.025, 0.25, 0.5, 0.75, and 0.095. The colors represent the different spider mite lines measured on bean (B), cucumber (C) and tomato (T) after 0 and 150 days on the respective host plant. The letters indicate significant differences and are corrected for multiple comparisons.

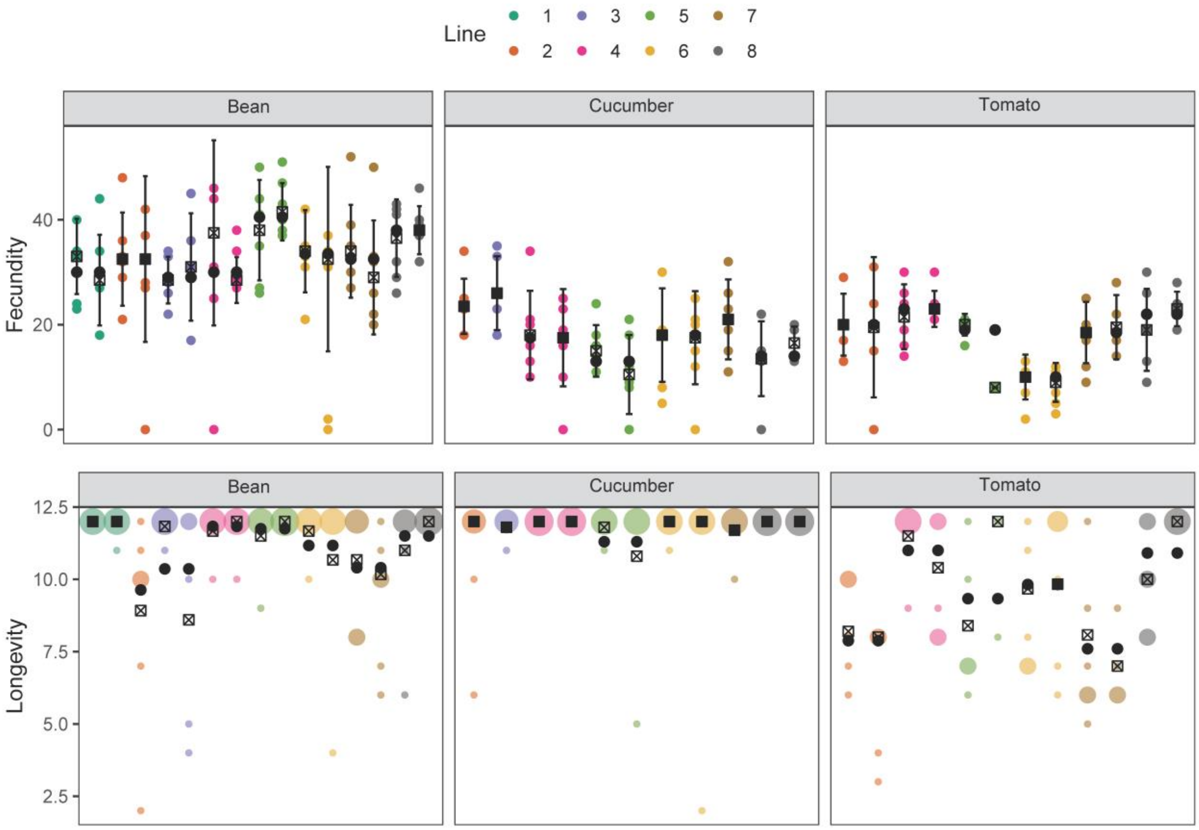

Figure S8: Fecundity and longevity for the different spider mite lines and replicates at day 150.

The *y*-axis shows the number of eggs after six days (fecundity) and the number of days the mites were surviving during the performance tests (longevity). The *x*-axis indicates the different lines which can be distinguished with the different colors. The squares are the median fecundity (upper) or mean longevity (lower) per replicate, while the black dots indicate the median fecundity or mean longevity for the different spider mite lines. The error bars show the standard deviation.

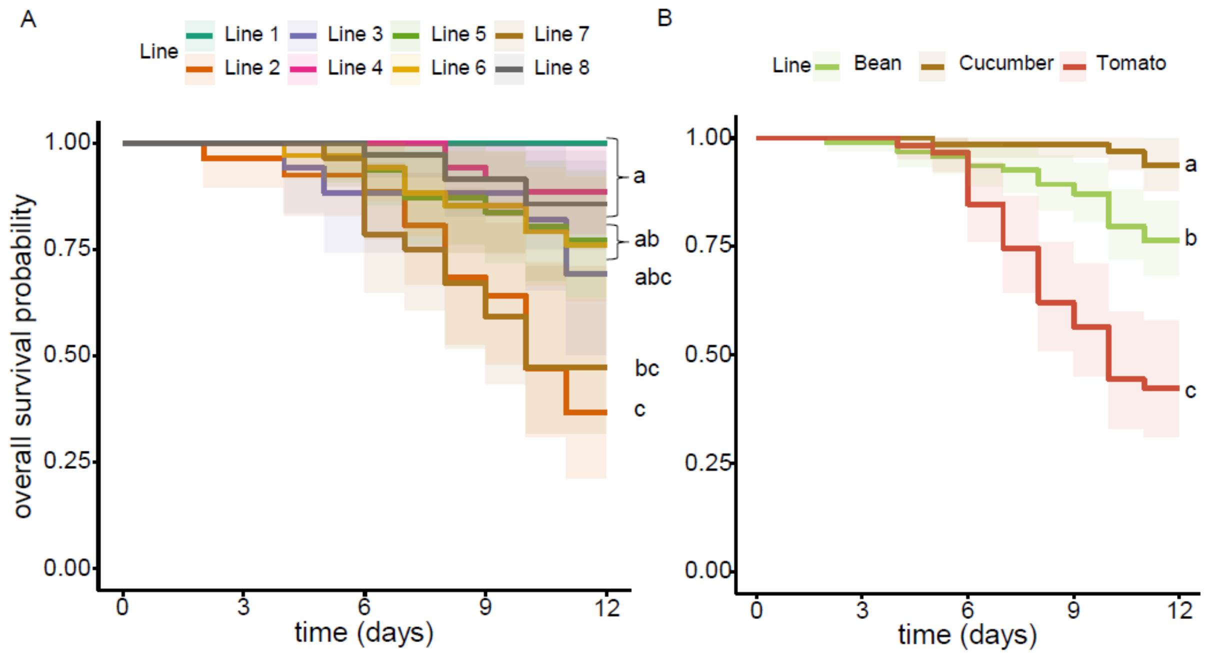

Figure S9: Survival probability of the spider mites for the different (A) spider mite lines and (B) plant species at day 150.

The *y*-axis shows the overall survival probability per population in time on the *x*-axis. The letters indicate the significance differences among (A) lines and (B) plant species. Spider mite lines 1, 4, and 8 have a higher survival probability than lines 2 (p value = 0.007, p value < 0.001, and p value = 0.001 respectively) and 7 (p value = 0.016, p value = 0.002, and p value = 0.006 respectively), with spider mite line 2 also being lower than lines 5 (p value = 0.016) and 6 (p value = 0.016). Mites on cucumber live longer than those on bean (p value = 0.004) and tomato (p value < 0.001), with mites on tomato having an even lower survival than those on bean (p value < 0.001). *P* values are obtained via pairwise comparisons using Log-Rank test and the Benjamini-Hochberg adjustment method.

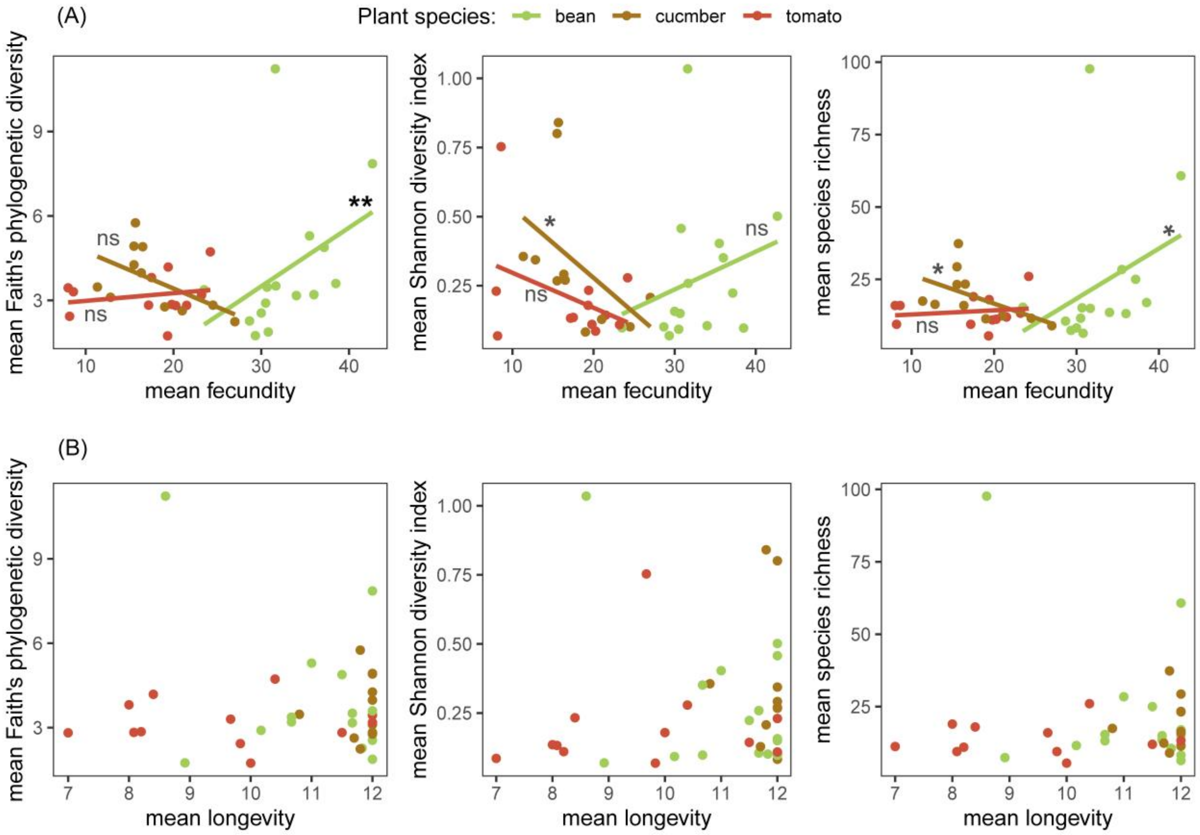

Figure S10: Correlations between alpha diversity metrics and fecundity/longevity (day 150).

The x-axis represents the (A) mean fecundity and (B) mean longevity per unit. The y-axis shows the different alpha metrics (i.e., Faith's phylogenetic diversity, Shannon diversity index, and species richness). The colors indicate the different plant species on which spider mite populations were reared. The asterisks show the significant slopes (* = *p* value between 0.05 and 0.01, ** = *p* value between 0.01 and 0.001).

TABLES

Table S1: Overview of the total number of microbiome samples in the rarefied datasets. The data is provided per genetically depleted line (including the two mixed populations; 7 and 8) and per plant species (bean, cucumber, and tomato). The percentages show the relative number of samples taken from a certain plant species compared to the total number of samples.

Line	1	2	3	4	5	6	7	8	Total
Bean	9	9	8	10	7	10	10	10	73 (49.3%
Cucumber	0	5	5	8	5	10	5	8	46 (31.1%)
Tomato	0	4	0	5	2	7	6	5	29 (19.6%)
Total	9	18	13	23	14	27	21	23	148

Table S2: Relationship between spider mite fitness proxies and microbiome community structure, using Procrustes and Mantel tests.

The distance matrices used for both the Procrustes and the Mantel test measure the differences between the microbiome composition of the different spider mite lines on their host plant (bean, cucumber, or tomato) and the differences between the fecundity, longevity, or both fecundity and longevity at the last time point (150 days). The significant results are visualized in grey. For the microbiome distance table, the unweighted UniFrac was chosen, while for the performance table two different distance measures were used (i.e. Euclidian and Manhattan). The Mantel test was done with 9999 permutations based on the Pearson method.

	Fecundity						Longevity						Fecundity and Longevity					
	bean		cucumber		tomato		bean		cucumber		tomato		bean		cucumber		tomato	
	st.	p-v.	st.	p-v.	st.	p-v.	st.	p-v.	st.	p-v.	st.	p-v.	st.	p-v.	st.	p-v.	st.	p-v.
Euclidian	**0.72**	**0.039**	0.68	0.051	0.77	0.111	0.876	0.596	0.85	0.551	**0.65**	**0.025**	**0.67**	**0.025**	0.68	0.051	0.71	0.071
	**0.73**	**0.044**	0.69	0.101	0.78	0.116	0.857	0.367	0.87	0.783	**0.65**	**0.004**	**0.68**	**0.028**	0.69	0.101	0.71	0.074
	0.73	0.052	0.68	0.071	**0.75**	**0.045**	0.853	0.335	0.86	0.671	0.73	0.097	**0.68**	**0.028**	0.68	0.072	**0.69**	**0.037**
	**0.73**	**0.040**	0.72	0.225	0.81	0.324	0.876	0.635	0.86	0.626	**0.71**	**0.044**	**0.68**	**0.032**	0.72	0.220	0.76	0.233
	0.73	0.056	0.74	0.353	0.79	0.163	0.855	0.358	0.86	0.749	**0.70**	**0.032**	**0.69**	**0.044**	0.73	0.352	0.73	0.089
Manhattan	**0.72**	**0.039**	0.68	0.051	0.77	0.111	0.876	0.596	0.85	0.551	**0.65**	**0.025**	**0.66**	**0.033**	**0.66**	**0.046**	**0.67**	**0.043**
	**0.73**	**0.044**	0.69	0.101	0.78	0.116	0.857	0.367	0.87	0.783	**0.65**	**0.004**	**0.66**	**0.028**	0.68	0.104	**0.68**	**0.045**
	0.73	0.052	0.68	0.071	**0.75**	**0.045**	0.853	0.335	0.86	0.671	0.73	0.097	**0.66**	**0.036**	0.67	0.069	**0.67**	**0.033**
	**0.73**	**0.040**	0.72	0.225	0.81	0.324	0.876	0.635	0.86	0.626	**0.71**	**0.044**	**0.66**	**0.034**	0.71	0.204	0.73	0.192
	0.73	0.056	0.74	0.353	0.79	0.163	0.855	0.358	0.86	0.749	**0.70**	**0.032**	**0.66**	**0.041**	0.72	0.344	0.70	0.057
Euclidian	0.27	0.062	-0.20	0.909	0.11	0.188	0.159	0.142	-0.22	0.841	**0.31**	**0.019**	**0.30**	**0.041**	-0.20	0.909	0.14	0.133
	0.24	0.084	-0.13	0.773	0.09	0.287	0.217	0.090	-0.30	0.932	**0.33**	**0.013**	0.29	0.054	-0.13	0.777	0.13	0.226
	0.23	0.095	-0.30	0.974	0.21	0.077	0.240	0.070	-0.29	0.853	0.12	0.183	0.27	0.057	-0.30	0.975	0.21	0.073
	0.24	0.087	-0.18	0.896	0.03	0.430	0.126	0.196	-0.16	0.814	0.16	0.117	0.27	0.065	-0.18	0.898	0.03	0.412
	0.19	0.127	-0.31	0.978	0.06	0.333	0.260	0.052	-0.31	0.917	0.18	0.097	0.24	0.084	-0.32	0.978	0.07	0.309
Manhattan	0.27	0.062	-0.20	0.909	0.11	0.188	0.159	0.142	-0.22	0.841	**0.31**	**0.019**	**0.31**	**0.036**	-0.22	0.926	0.18	0.083
	0.24	0.084	-0.13	0.773	0.09	0.287	0.217	0.090	-0.30	0.932	**0.33**	**0.013**	**0.30**	**0.042**	-0.16	0.819	0.17	0.137
	0.23	0.095	-0.30	0.974	0.21	0.077	0.240	0.070	-0.29	0.853	0.12	0.183	**0.29**	**0.045**	-0.32	0.980	0.23	0.051
	0.24	0.087	-0.18	0.896	0.03	0.430	0.126	0.196	-0.16	0.814	0.16	0.117	0.27	0.059	-0.19	0.909	0.07	0.331
	0.19	0.127	-0.31	0.978	0.06	0.333	0.260	0.052	-0.31	0.917	0.18	0.097	0.26	0.063	-0.34	0.983	0.10	0.234

Table S3: Entire overview of all ASVs per plant and spider mite line (TableS3.xlsx)

Table S4: Model selection for phylogenetic distance.

The removal of the non-significant independent variables is given per rarefied dataset (seed 1, seed 2, etc.). The maximal model included the plant species, the spider mite line, and their interaction as independent variables, while the unit was considered as a random effect. Only the spider mite line influenced the phylogenetic distance in all tested rarefied datasets.

Removal of non-significant variables<br>Maximal model: phylogenetic distance ~ plant species * host spider mite line + (1|unit)								
		df	AIC	logLik	Chisq	Chi df	Pr(>Chisq)	
seed 1	Plant species * Line	23	220.9	-87.4	13.6	11	0.259	
	Plant species + Line	12	212.5	-94.2	1.9	2	0.378	
	**Line**	10	210.4	-95.2	13.8	7	0.055	.
	without independent variables	3	210.2	-102.1				
seed 2	Plant species * Line	23	237.5	-95.7	16.3	11	0.130	
	Plant species + Line	12	231.8	-103.9	3.4	2	0.183	
	**Line**	10	231.2	-105.6	13.9	7	0.052	.
	without independent variables	3	231.1	-112.6				
seed 3	Plant species * Line	23	215.5	-84.7	12.8	11	0.306	
	Plant species + Line	12	206.3	-91.1	2.0	2	0.370	
	**Line**	10	204.3	-92.1	16.1	7	0.025	*
	without independent variables	3	206.3	-100.2				
seed 4	Plant species * Line	23	210.0	-82.0	13.3	11	0.275	
	Plant species + Line	12	201.3	-88.6	2.0	2	0.377	
	**Line**	10	199.2	-89.6	15.2	7	0.033	*
	without independent variables	3	200.4	-97.2				
seed 5	Plant species * Line	23	217.3	-85.6	14.8	11	0.193	
	Plant species + Line	12	210.1	-93.0	1.6	2	0.438	
	**Line**	10	207.7	-93.9	14.6	7	0.042	*
	without independent variables	3	208.3	-101.1				

Table S5: Model summary and significant pairwise comparisons for phylogenetic distance.

The intervals indicate the maximum and minimum value obtained with the different seeds.

Model summary for phylogenetic distance					
	estimate	std. error	*z* value	Pr(>|z|)	
(Intercept) (Line 1)	[0.70; 0.75]	[0.20; 0.21]	[3.39; 3.79]	<0.001	***
Line 2	[0.14; 0.20]	[0.24; 0.25]	[0.56; 0.78]	[0.433; 0.580]	
Line 3	[0.46; 0.50]	[0.25; 0.27]	[1.76; 1.91]	[0.056; 0.080]	./*
Line 4	[0.46; 0.55]	[0.23; 0.24]	[1.93; 2.26]	[0.024; 0.050]	*/**
Line 5	[0.80; 0.84]	[0.24; 0.26]	[3.19; 3.38]	<0.001	***
Line 6	[0.40; 0.46]	[0.23; 0.24]	[1.65; 2.02]	[0.043; 0.100]	./*
Line 7	[0.17; 0.28]	[0.23; 0.25]	[0.71; 1.11]	[0.265; 0.480]	
Line 8	[0.48; 0.52]	[0.23; 0.24]	[2.01; 2.25]	[0.024; 0.040]	*

Significant pairwise comparisons for phylogenetic distance					
Contrast	estimate	std. error	df	*t* ratio	*p* value
Line 1 – Line 5	[-0.84; -0.8]	[0.24; 0.26]	138	[-3.38; -3.19]	[0.021; 0.037] *
Line 2 – Line 5	[-0.66; -0.63]	[0.2; 0.21]	138	[-3.33; -3.09]	[0.024; 0.048] *
Line 5 – Line 7	[0.57; 0.65]	[0.19; 0.2]	138	[2.81; 3.42]	[0.018; 0.102] ./*

Table S6: Model selection for Shannon diversity.

The removal of the non-significant independent variables is given per rarefied dataset (seed 1, seed 2, etc.). The maximal model included the plant species, the spider mite line, and their interaction as independent variables, while the different units within their batch were considered as a random effect. Both the spider mite line and the host plant species influenced the Shannon diversity (but not their interaction) in all tested rarefied datasets.

Removal of non-significant variables<br>Maximal model: Shannon diversity ~ plant species * host spider mite line + (1|batch/unit)								
		df	AIC	logLik	Chisq	Chi df	Pr(>Chisq)	
seed 1	Plant species * Line	24	438.9	-195.5	7.9	11	0.726	
	Plant species + Line	13	424.8	-199.4	3.7	2	0.161	
	**Line**	11	424.5	-201.2	14.4	7	0.045	*
	without independent variables	4	424.8	-208.4				
seed 2	Plant species * Line	24	437.7	-194.8	7.7	11	0.737	
	Plant species + Line	13	423.4	-198.7	4.0	2	0.137	
	**Line**	11	423.4	-200.7	14.8	7	0.039	*
	without independent variables	4	424.2	-208.1				
seed 3	Plant species * Line	24	432.1	-192.1	7.5	11	0.755	
	Plant species + Line	13	417.7	-195.8	3.9	2	0.143	
	**Line**	11	417.6	-197.8	14.6	7	0.041	*
	without independent variables	4	418.2	-205.1				
seed 4	Plant species * Line	24	432.8	-192.4	7.5	11	0.755	
	Plant species + Line	13	418.4	-196.2	3.8	2	0.150	
	**Line**	11	418.2	-198.1	14.6	7	0.041	*
	without independent variables	4.0	418.8	-205.4				
seed 5	Plant species * Line	24	432.2	-192.1	7.4	11	0.769	
	Plant species + Line	13	417.6	-195.8	3.9	2	0.140	
	**Line**	11	417.5	-197.8	14.7	7	0.040	*
	without independent variables	4	418.2	-205.1				

Table S7: Model summary and significant pairwise comparisons for Shannon diversity.

The intervals indicate the maximum and minimum value obtained with the different seeds.

Model summary for Shannon diversity					
	estimate	std. error	z value	Pr(>|z|)	
(Intercept) (Line 1)	[-1.45; -1.44]	[0.44; 0.45]	[-3.27; -3.18]	<0.001	***
Line 2	[-1.28; -1.26]	[0.54; 0.55]	[-2.36; -2.32]	[0.018; 0.020]	*
Line 3	[-0.16; -0.13]	[0.58; 0.59]	[-0.28; -0.22]	[0.776; 0.830]	
Line 4	[-0.38; -0.35]	[0.52; 0.53]	[-0.72; -0.68]	[0.473; 0.500]	
Line 5	[0.40; 0.42]	[0.54; 0.55]	[0.74; 0.77]	[0.440; 0.460]	
Line 6	[-0.55; -0.50]	[0.51; 0.52]	[-1.06; -0.98]	[0.290; 0.330]	
Line 7	[-0.76; -0.73]	[0.53; 0.54]	[-1.43; -1.37]	[0.154; 0.170]	
Line 8	[-0.45; -0.39]	[0.52; 0.53]	[-0.85; -0.75]	[0.393; 0.450]	
**Significant pairwise comparisons for Shannon diversity**					
Contrast	estimate	std. error	df	*t* ratio	*p* value
Line 2 - Line 5	[-1.69; -1.67]	[0.43; 0.44]	137	[-3.9; -3.81]	[0.004; 0.005] **

Table S8: Model selection for species richness.

The removal of the non-significant independent variables is given per rarefied dataset (seed 1, seed 2, etc.). The maximal model included the plant species, the spider mite line, and their interaction as independent variables, while the unit nested in its batch was considered as a random effect. Both the spider mite line and the host plant species influenced the Shannon diversity (but not their interaction) in all tested rarefied datasets.

Removal of non-significant variables<br>Maximal model: species richness ~ plant species * host spider mite line + (1|batch/unit)								
		df	AIC	logLik	Chisq	Chi df	Pr(>Chisq)	
seed 1	Plant species * Line	24	1083.3	-517.7	14.5	11	0.205	
	Plant species + Line	13	1075.8	-524.9	2.6	2	0.271	
	**Line**	11	1074.5	-526.2	16.2	7	0.024	*
	without independent variables	4	1076.6	-534.3				
seed 2	Plant species * Line	24	1079.3	-515.7	15.9	11	0.146	
	Plant species + Line	13	1073.2	-523.6	2.8	2	0.242	
	**Line**	11	1072.0	-525.0	15.5	7	0.030	*
	without independent variables	4	1073.6	-532.8				
seed 3	Plant species * Line	24	1086.2	-519.1	13.6	11	0.254	
	Plant species + Line	13	1077.8	-525.9	2.6	2	0.279	
	**Line**	11	1076.4	-527.2	15.7	7	0.028	*
	without independent variables	4	1078.1	-535.1				
seed 4	Plant species * Line	24	1080.7	-516.3	14.5	11	0.205	
	Plant species + Line	13	1073.2	-523.6	2.4	2	0.306	
	**Line**	11	1071.5	-524.8	15.9	7	0.026	*
	without independent variables	4.0	1073.4	-532.7				
seed 5	Plant species * Line	24	1086.2	-519.1	15.6	11	0.157	
	Plant species + Line	13	1079.8	-526.9	2.3	2	0.319	
	**Line**	11	1078.1	-528.1	15.6	7	0.029	*
	without independent variables	4	1079.7	-535.9				

Table S9: Model summary and significant pairwise comparisons for species richness.

The intervals indicate the maximum and minimum value obtained with the different seeds.

Model summary for species richness					
	estimate	std. error	z value	Pr(>|z|)	
(Intercept) (Line 1)	[1.95; 2.02]	[0.32; 0.33]	[5.99; 6.36]	<0.001	***
Line 2	[0.39; 0.46]	[0.38; 0.39]	[1.02; 1.19]	[0.235; 0.31]	
Line 3	[1.04; 1.10]	[0.40; 0.41]	[2.54; 2.69]	[0.007; 0.01]	*/**
Line 4	[0.81; 0.90]	[0.37; 0.38]	[2.21; 2.38]	[0.017; 0.03]	*
Line 5	[1.38; 1.45]	[0.38; 0.39]	[3.64; 3.73]	<0.001	***
Line 6	[0.75; 0.80]	[0.36; 0.37]	[2.05; 2.15]	[0.031; 0.04]	*
Line 7	[0.44; 0.53]	[0.37; 0.39]	[1.16; 1.37]	[0.171; 0.25]	
Line 8	[0.81; 0.90]	[0.37; 0.38]	[2.21; 2.41]	[0.016; 0.03]	*

Significant pairwise comparisons for species richness					
Contrast	estimate	std. error	df	*t* ratio	*p* value
Line 1 - Line 5	[-1.45; -1.38]	[0.38; 0.39]	137	[-3.73; -3.64]	[0.007; 0.009] **
Line 2 - Line 5	[-1.01; -0.99]	[0.30; 0.30]	137	[-3.38; -3.26]	[0.020; 0.030] *
Line 5 - Line 7	[0.91; 0.98]	[0.29; 0.3]	137	[3.09; 3.36]	[0.020; 0.050] *

Table S10: Output libshuff method in Mothur, using the Cramer-von Mises test statistic. The test statistic is given with its significance value for the different plant species, batches, and seeds. We used the unweighted UniFrac distance to create the distance matrices. The values in bold are below 0.05 and significant values after Bonferroni's correction are bold and underlined. The grey lines are comparisons for which more than one rarefied dataset revealed a value below 0.05.

			seed 1		seed 2		seed 3		seed 4		seed 5	
		Comparison	dCXY Score	Sign.	dCXY Score	Sign.	dCXY Score	Sign.	dCXY Score	Sign.	dCXY Score	Sign.
Bean	batch 1	Line 1.1-Line 1.2	0.016	0.556	0.006	1.000	0.013	0.556	0.019	0.444	0.019	0.778
		Line 1.2-Line 1.1	0.054	0.778	0.054	0.444	0.029	0.667	0.033	0.556	0.038	0.556
		Line 1.1-Line 2.1	0.060	0.111	0.064	**<0.001**	0.047	0.111	0.049	0.111	0.036	**<0.001**
		Line 2.1-Line 1.1	0.033	0.778	0.046	0.889	0.048	0.667	0.033	0.778	0.018	0.889
		Line 1.1-Line 2.2	0.035	0.400	0.044	0.400	0.044	0.200	0.022	0.600	0.052	0.100
		Line 2.2-Line 1.1	0.052	0.200	0.043	0.200	0.052	0.300	0.030	0.400	0.028	0.600
		Line 1.1-Line 3.1	0.073	0.200	0.076	**<0.001**	0.039	0.400	0.063	0.200	0.051	0.200
		Line 3.1-Line 1.1	0.019	0.400	0.020	0.400	0.024	0.200	0.019	0.600	0.024	0.500
		Line 1.1-Line 3.2	0.376	**<0.001**	0.365	**<0.001**	0.350	**<0.001**	0.347	0.125	0.347	**<0.001**
		Line 3.2-Line 1.1	0.223	**<0.001**	0.227	0.125	0.235	0.125	0.229	**<0.001**	0.193	**<0.001**
		Line 1.1-Line 7.1	0.024	0.400	0.013	0.900	0.020	0.400	0.022	0.500	0.016	1.000
		Line 7.1-Line 1.1	0.019	0.700	0.023	0.800	0.018	0.600	0.014	0.900	0.030	0.700
		Line 1.1-Line 7.2	0.060	0.300	0.052	0.300	0.041	0.100	0.039	0.400	0.047	0.300
		Line 7.2-Line 1.1	0.028	0.500	0.017	0.800	0.014	0.900	0.014	0.900	0.041	0.600
		Line 1.2-Line 2.1	0.045	**<0.001**	0.021	0.750	0.015	0.750	0.024	0.500	0.046	**<0.001**
		Line 2.1-Line 1.2	0.011	1.000	0.022	0.500	0.015	1.000	0.018	0.750	0.016	1.000
		Line 1.2-Line 2.2	0.022	0.889	0.007	1.000	0.031	0.667	0.036	0.444	0.049	0.222
		Line 2.2-Line 1.2	0.021	0.667	0.050	0.111	0.054	0.222	0.018	0.778	0.032	0.222
		Line 1.2-Line 3.1	0.049	0.444	0.023	0.889	0.045	0.667	0.047	0.556	0.046	0.556
		Line 3.1-Line 1.2	0.062	**<0.001**	0.027	0.889	0.072	0.111	0.040	**<0.001**	0.048	0.111
		Line 1.2-Line 3.2	0.262	0.143	0.236	**<0.001**	0.280	**<0.001**	0.311	**<0.001**	0.327	0.143
		Line 3.2-Line 1.2	0.135	0.286	0.138	0.143	0.146	0.286	0.172	0.286	0.099	0.286
		Line 1.2-Line 7.1	0.048	0.333	0.024	0.889	0.014	0.556	0.014	0.778	0.026	0.667
		Line 7.1-Line 1.2	0.062	0.111	0.028	0.445	0.066	0.111	0.032	0.111	0.034	0.333
		Line 1.2-Line 7.2	0.028	0.667	0.017	1.000	0.033	0.667	0.028	0.667	0.041	0.444
		Line 7.2-Line 1.2	0.011	1.000	0.011	1.000	0.028	0.667	0.012	0.778	0.018	0.667
		Line 2.1-Line 2.2	0.032	0.667	0.024	0.778	0.031	0.778	0.026	0.667	0.009	1.000
		Line 2.2-Line 2.1	0.026	0.333	0.038	0.222	0.031	0.222	0.013	0.667	0.042	0.222
		Line 2.1-Line 3.1	0.011	1.000	0.013	0.778	0.018	0.667	0.010	0.778	0.011	1.000
		Line 3.1-Line 2.1	0.028	0.444	0.017	0.556	0.019	0.556	0.007	1.000	0.023	0.222
		Line 2.1-Line 3.2	0.199	0.143	0.209	0.143	0.201	0.143	0.229	**<0.001**	0.211	0.143
		Line 3.2-Line 2.1	0.126	0.571	0.144	0.143	0.148	0.286	0.147	**<0.001**	0.117	0.286
		Line 2.1-Line 7.1	0.008	1.000	0.014	1.000	0.018	0.667	0.012	0.889	0.006	0.889
		Line 7.1-Line 2.1	0.023	0.222	0.028	0.333	0.029	0.111	0.031	0.333	0.013	0.667
		Line 2.1-Line 7.2	0.026	0.556	0.031	0.667	0.033	0.222	0.021	0.889	0.011	1.000
		Line 7.2-Line 2.1	0.008	1.000	0.011	0.889	0.028	0.333	0.010	1.000	0.020	0.444
		Line 2.2-Line 3.1	0.027	0.400	0.029	0.400	0.039	0.500	0.007	1.000	0.027	0.200
		Line 3.1-Line 2.2	0.013	0.600	0.004	0.800	0.036	0.300	0.028	0.100	0.012	0.500
		Line 2.2-Line 3.2	0.149	0.125	0.132	**<0.001**	0.109	**<0.001**	0.189	**<0.001**	0.163	**<0.001**
		Line 3.2-Line 2.2	0.182	0.125	0.182	0.125	0.184	**<0.001**	0.185	0.250	0.155	0.250
		Line 2.2-Line 7.1	0.044	0.100	0.047	0.200	0.058	0.100	0.022	0.200	0.046	0.100
		Line 7.1-Line 2.2	0.008	0.900	0.013	0.800	0.007	0.800	0.010	0.900	0.015	0.900
		Line 2.2-Line 7.2	0.065	0.300	0.080	0.200	0.062	0.200	0.025	0.800	0.071	**<0.001**
		Line 7.2-Line 2.2	0.022	0.800	0.022	0.700	0.037	0.600	0.023	0.700	0.010	1.000
		Line 3.1-Line 3.2	0.296	**<0.001**	0.232	0.125	0.284	**<0.001**	0.252	0.125	0.288	**<0.001**
		Line 3.2-Line 3.1	0.136	0.250	0.156	**<0.001**	0.175	0.125	0.159	0.125	0.147	0.125
		Line 3.1-Line 7.1	0.062	0.200	0.030	0.600	0.058	0.200	0.033	0.600	0.066	0.100
		Line 7.1-Line 3.1	0.029	0.400	0.032	0.400	0.041	0.200	0.034	0.500	0.034	0.400
		Line 3.1-Line 7.2	0.016	0.700	0.024	0.500	0.015	0.900	0.014	0.800	0.022	0.200
		Line 7.2-Line 3.1	0.013	0.900	0.026	0.600	0.018	0.500	0.014	0.800	0.012	0.700
		Line 3.2-Line 7.1	0.103	0.250	0.111	0.375	0.114	0.375	0.112	0.250	0.112	0.125
		Line 7.1-Line 3.2	0.172	**<0.001**	0.194	0.125	0.168	0.125	0.176	**<0.001**	0.178	**<0.001**
		Line 3.2-Line 7.2	0.148	0.500	0.151	0.375	0.151	0.375	0.151	0.375	0.129	0.625
		Line 7.2-Line 3.2	0.172	**<0.001**	0.162	**<0.001**	0.198	0.125	0.186	0.125	0.240	**<0.001**
		Line 7.1-Line 7.2	0.006	0.900	0.006	0.800	0.023	0.300	0.013	0.700	0.020	0.700
		Line 7.2-Line 7.1	0.013	0.600	0.009	0.900	0.016	0.700	0.012	0.900	0.021	0.400
	batch 2	Line 4.1-Line 4.2	0.016	0.400	0.010	0.800	0.034	0.200	0.019	0.400	0.034	**<0.001**
		Line 4.2-Line 4.1	0.017	0.200	0.007	0.900	0.010	0.500	0.008	0.600	0.018	0.600
		Line 4.1-Line 5.1	0.034	0.250	0.031	0.250	0.038	0.500	0.050	0.125	0.023	0.250
		Line 5.1-Line 4.1	0.029	0.625	0.025	0.500	0.042	0.250	0.020	0.375	0.026	0.500
		Line 4.1-Line 5.2	0.046	0.111	0.055	0.111	0.067	**<0.001**	0.084	**<0.001**	0.060	**<0.001**
		Line 5.2-Line 4.1	0.018	0.778	0.021	0.778	0.020	0.222	0.032	0.445	0.026	0.667
		Line 4.1-Line 6.1	0.034	0.500	0.020	0.500	0.036	0.400	0.027	0.500	0.040	0.400
		Line 6.1-Line 4.1	0.006	0.900	0.006	0.800	0.010	0.800	0.007	0.900	0.008	1.000
		Line 4.1-Line 6.2	0.043	0.100	0.025	0.500	0.014	0.900	0.019	0.600	0.039	0.200
		Line 6.2-Line 4.1	0.051	0.200	0.043	0.100	0.059	0.200	0.040	0.200	0.024	0.600
		Line 4.1-Line 8.1	0.019	0.400	0.018	0.300	0.030	0.100	0.026	0.200	0.011	0.500
		Line 8.1-Line 4.1	0.009	0.800	0.008	0.600	0.011	0.400	0.007	0.800	0.015	0.400
		Line 4.1-Line 8.2	0.013	0.800	0.008	0.700	0.015	0.500	0.026	0.300	0.009	0.800
		Line 8.2-Line 4.1	0.017	0.700	0.003	0.900	0.032	0.100	0.014	0.600	0.038	0.100
		Line 4.2-Line 5.1	0.031	0.375	0.021	0.500	0.035	0.125	0.055	0.125	0.029	0.250
		Line 5.1-Line 4.2	0.029	0.375	0.023	0.750	0.038	0.625	0.027	0.625	0.031	0.375
		Line 4.2-Line 5.2	0.088	**<0.001**	0.102	0.111	0.056	0.111	0.096	0.111	0.074	0.111
		Line 5.2-Line 4.2	0.012	0.889	0.012	1.000	0.017	0.667	0.012	0.889	0.014	0.667
		Line 4.2-Line 6.1	0.018	0.500	0.008	0.900	0.014	0.600	0.005	0.800	0.024	0.400
		Line 6.1-Line 4.2	0.005	1.000	0.007	0.600	0.010	0.900	0.020	0.400	0.011	0.700
		Line 4.2-Line 6.2	0.009	0.800	0.009	0.900	0.008	0.800	0.006	1.000	0.019	0.400
		Line 6.2-Line 4.2	0.022	0.300	0.025	0.400	0.032	0.200	0.038	0.200	0.015	0.600
		Line 4.2-Line 8.1	0.013	0.100	0.015	0.600	0.010	0.700	0.009	0.600	0.008	0.300
		Line 8.1-Line 4.2	0.010	0.600	0.009	0.700	0.006	0.700	0.016	0.300	0.014	0.100
		Line 4.2-Line 8.2	0.015	0.400	0.009	0.800	0.003	0.800	0.007	0.900	0.006	0.600
		Line 8.2-Line 4.2	0.053	0.100	0.038	0.100	0.083	**<0.001**	0.024	0.100	0.028	0.100

		**Line 5.1-Line 5.2**	0.027	0.714	0.044	0.714	0.032	0.572	0.022	0.714	0.053	0.857
		**Line 5.2-Line 5.1**	0.013	0.714	0.016	0.714	0.024	0.571	0.016	0.714	0.020	0.714
		**Line 5.1-Line 6.1**	0.031	0.875	0.028	0.750	0.051	0.625	0.037	0.625	0.033	0.750
		**Line 6.1-Line 5.1**	0.038	0.125	0.017	0.250	0.045	0.250	0.022	0.375	0.015	0.625
		**Line 5.1-Line 6.2**	0.027	1.000	0.029	1.000	0.033	1.000	0.037	0.875	0.034	0.875
		**Line 6.2-Line 5.1**	0.055	0.125	0.053	0.125	0.056	0.250	0.060	0.125	0.029	**<0.001**
		**Line 5.1-Line 8.1**	0.026	0.500	0.025	0.625	0.075	**<0.001**	0.028	0.500	0.025	0.625
		**Line 8.1-Line 5.1**	0.011	0.375	0.006	0.875	0.017	0.500	0.008	0.750	0.004	1.000
		**Line 5.1-Line 8.2**	0.026	0.625	0.020	0.875	0.035	0.500	0.019	0.625	0.027	0.625
		**Line 8.2-Line 5.1**	0.006	0.750	0.004	0.875	0.020	**<0.001**	0.006	0.875	0.013	0.750
		**Line 5.2-Line 6.1**	0.016	1.000	0.016	0.889	0.025	0.889	0.016	0.889	0.020	0.889
		**Line 6.1-Line 5.2**	0.079	**<0.001**	0.102	0.111	0.063	0.111	0.064	**<0.001**	0.067	0.111
		**Line 5.2-Line 6.2**	0.019	1.000	0.023	1.000	0.025	1.000	0.021	1.000	0.021	0.778
		**Line 6.2-Line 5.2**	0.040	0.222	0.047	0.222	0.031	0.222	0.044	0.222	0.029	0.111
		**Line 5.2-Line 8.1**	0.020	0.556	0.020	0.778	0.032	0.222	0.020	0.778	0.025	0.333
		**Line 8.1-Line 5.2**	0.046	0.111	0.038	**<0.001**	0.058	0.111	0.046	0.111	0.040	0.111
		**Line 5.2-Line 8.2**	0.019	0.889	0.024	0.889	0.021	0.444	0.016	0.778	0.014	0.667
		**Line 8.2-Line 5.2**	0.049	**<0.001**	0.056	0.111	0.060	0.111	0.068	0.111	0.059	0.111
		**Line 6.1-Line 6.2**	0.013	0.500	0.026	0.400	0.015	0.600	0.013	0.600	0.013	0.800
		**Line 6.2-Line 6.1**	0.030	0.500	0.037	0.500	0.026	0.400	0.020	0.500	0.016	0.600
		**Line 6.1-Line 8.1**	0.013	0.600	0.016	0.200	0.020	0.500	0.018	0.400	0.003	0.900
		**Line 8.1-Line 6.1**	0.013	0.600	0.013	0.400	0.007	0.700	0.008	0.800	0.021	0.500
		**Line 6.1-Line 8.2**	0.017	0.500	0.008	0.600	0.018	0.700	0.009	0.800	0.010	0.800
		**Line 8.2-Line 6.1**	0.045	0.300	0.013	0.700	0.058	0.100	0.024	0.600	0.036	0.400
		**Line 6.2-Line 8.1**	0.046	**<0.001**	0.048	**<0.001**	0.042	0.200	0.050	0.100	0.026	0.400
		**Line 8.1-Line 6.2**	0.010	1.000	0.007	1.000	0.006	1.000	0.004	1.000	0.007	0.800
		**Line 6.2-Line 8.2**	0.031	0.100	0.019	0.500	0.022	0.100	0.030	**<0.001**	0.011	0.800
		**Line 8.2-Line 6.2**	0.029	0.500	0.028	0.400	0.030	0.300	0.014	0.800	0.014	0.400
		**Line 8.1-Line 8.2**	0.013	0.300	0.007	0.700	0.004	1.000	0.014	0.500	0.014	0.400
		**Line 8.2-Line 8.1**	0.014	0.300	0.006	0.800	0.026	0.100	0.012	0.600	0.020	0.400
**Cucumber**	**batch 1**	**Line 2.1-Line 3.1**	0.114	0.100	0.097	**<0.001**	0.059	**<0.001**	0.013	0.700	0.106	**<0.001**
		**Line 3.1-Line 2.1**	0.073	0.300	0.057	0.400	0.034	0.400	0.054	0.300	0.042	0.500
		**Line 2.1-Line 7.1**	0.044	0.400	0.072	0.200	0.068	**<0.001**	0.016	0.700	0.077	**<0.001**
		**Line 7.1-Line 2.1**	0.039	0.400	0.069	0.200	0.017	0.700	0.054	0.200	0.046	0.300
		**Line 3.1-Line 7.1**	0.033	0.900	0.029	0.900	0.042	0.600	0.036	0.600	0.024	0.700
		**Line 7.1-Line 3.1**	0.066	0.100	0.060	0.100	0.036	0.200	0.059	0.200	0.055	0.100
	**batch 2**	**Line 4.1-Line 4.2**	0.037	0.125	0.016	0.625	0.032	0.250	0.019	0.250	0.021	0.250
		**Line 4.2-Line 4.1**	0.009	0.875	0.024	0.875	0.016	0.750	0.014	0.875	0.011	0.875
		**Line 4.1-Line 5.1**	0.021	0.625	0.010	0.500	0.013	0.625	0.013	0.500	0.006	1.000
		**Line 5.1-Line 4.1**	0.047	0.625	0.045	0.250	0.048	0.125	0.063	0.250	0.035	0.500
		**Line 4.1-Line 5.2**	0.027	0.286	0.011	1.000	0.021	0.571	0.011	0.429	0.011	1.000
		**Line 5.2-Line 4.1**	0.126	0.286	0.147	0.286	0.129	0.571	0.118	0.571	0.158	0.571
		**Line 4.1-Line 6.1**	0.012	0.900	0.006	1.000	0.009	0.800	0.013	0.500	0.007	0.900
		**Line 6.1-Line 4.1**	0.065	**<0.001**	0.062	0.100	0.026	0.100	0.048	0.100	0.067	0.100
		**Line 4.1-Line 6.2**	0.026	0.400	0.013	0.600	0.033	0.200	0.025	0.400	0.014	0.600
		**Line 6.2-Line 4.1**	0.010	0.800	0.006	0.900	0.008	0.800	0.003	0.900	0.012	0.400
		**Line 4.1-Line 8.1**	0.053	0.100	0.013	0.500	0.022	0.300	0.022	0.200	0.020	0.300
		**Line 8.1-Line 4.1**	0.018	0.600	0.016	0.500	0.016	0.800	0.052	0.100	0.054	**<0.001**
		**Line 4.1-Line 8.2**	0.049	0.125	0.040	0.375	0.041	0.125	0.030	0.375	0.024	0.375
		**Line 8.2-Line 4.1**	0.021	0.875	0.017	1.000	0.036	0.500	0.041	0.750	0.012	0.875
		**Line 4.2-Line 5.1**	0.006	0.833	0.056	0.333	0.009	1.000	0.013	0.833	0.012	0.833
		**Line 5.1-Line 4.2**	0.022	**<0.001**	0.020	0.500	0.019	0.667	0.026	0.333	0.023	0.167
		**Line 4.2-Line 5.2**	0.018	0.600	0.017	0.800	0.015	0.400	0.018	0.800	0.009	1.000
		**Line 5.2-Line 4.2**	0.089	0.400	0.090	0.200	0.099	0.400	0.063	0.600	0.108	0.200
		**Line 4.2-Line 6.1**	0.007	1.000	0.029	0.625	0.011	1.000	0.022	0.750	0.009	0.750
		**Line 6.1-Line 4.2**	0.023	0.125	0.021	0.500	0.009	0.375	0.031	0.125	0.025	0.250
		**Line 4.2-Line 6.2**	0.002	1.000	0.031	0.625	0.007	0.875	0.015	0.875	0.007	0.750
		**Line 6.2-Line 4.2**	0.013	0.375	0.016	0.375	0.012	0.625	0.006	1.000	0.015	**<0.001**
		**Line 4.2-Line 8.1**	0.030	0.625	0.013	1.000	0.027	0.875	0.004	0.875	0.010	1.000
		**Line 8.1-Line 4.2**	0.013	0.375	0.019	0.250	0.016	0.250	0.028	0.125	0.019	0.125
		**Line 4.2-Line 8.2**	0.034	0.167	0.012	0.833	0.029	0.167	0.006	0.833	0.017	0.667
		**Line 8.2-Line 4.2**	0.012	0.667	0.011	0.333	0.026	0.500	0.034	**<0.001**	0.011	0.500
		**Line 5.1-Line 5.2**	0.036	0.600	0.015	0.600	0.012	1.000	0.037	**<0.001**	0.011	1.000
		**Line 5.2-Line 5.1**	0.143	0.600	0.118	0.600	0.064	0.800	0.065	0.800	0.114	0.600
		**Line 5.1-Line 6.1**	0.035	0.500	0.008	0.875	0.024	0.500	0.037	0.750	0.008	1.000
		**Line 6.1-Line 5.1**	0.048	0.625	0.063	**<0.001**	0.028	0.500	0.060	0.250	0.042	0.125
		**Line 5.1-Line 6.2**	0.020	0.625	0.012	0.625	0.008	0.750	0.011	0.875	0.006	0.875
		**Line 6.2-Line 5.1**	0.006	1.000	0.007	0.875	0.007	0.625	0.010	0.625	0.016	0.125
		**Line 5.1-Line 8.1**	0.009	0.875	0.008	1.000	0.005	0.875	0.013	0.875	0.007	0.875
		**Line 8.1-Line 5.1**	0.017	0.250	0.018	0.250	0.008	0.500	0.025	0.125	0.016	0.375
		**Line 5.1-Line 8.2**	0.013	1.000	0.020	0.833	0.016	0.667	0.022	1.000	0.013	1.000
		**Line 8.2-Line 5.1**	0.019	0.833	0.017	0.667	0.026	0.667	0.030	0.333	0.016	0.667
		**Line 5.2-Line 6.1**	0.122	0.429	0.165	0.571	0.130	0.286	0.123	0.286	0.145	0.429
		**Line 6.1-Line 5.2**	0.034	0.714	0.045	0.572	0.022	1.000	0.057	0.429	0.035	0.571
		**Line 5.2-Line 6.2**	0.162	0.429	0.141	0.429	0.145	0.429	0.116	0.429	0.183	0.571
		**Line 6.2-Line 5.2**	0.014	0.857	0.017	0.714	0.033	0.572	0.014	0.714	0.014	0.857
		**Line 5.2-Line 8.1**	0.046	0.572	0.045	0.572	0.033	1.000	0.048	0.857	0.060	0.571
		**Line 8.1-Line 5.2**	0.047	0.571	0.019	0.857	0.041	0.286	0.046	0.286	0.046	0.286
		**Line 5.2-Line 8.2**	0.019	1.000	0.024	1.000	0.049	0.800	0.034	1.000	0.019	1.000
		**Line 8.2-Line 5.2**	0.009	0.800	0.008	1.000	0.042	0.600	0.047	0.600	0.011	0.800
		**Line 6.1-Line 6.2**	0.034	0.400	0.042	0.400	0.022	0.600	0.040	0.500	0.034	0.500
		**Line 6.2-Line 6.1**	0.012	0.800	0.015	0.700	0.018	0.700	0.012	0.800	0.009	0.700
		**Line 6.1-Line 8.1**	0.011	0.600	0.045	0.100	0.011	0.600	0.043	0.300	0.014	0.400
		**Line 8.1-Line 6.1**	0.041	0.100	0.034	0.300	0.034	0.100	0.085	**<0.001**	0.026	0.100
		**Line 6.1-Line 8.2**	0.008	0.625	0.012	0.875	0.003	0.875	0.013	0.750	0.025	0.750
		**Line 8.2-Line 6.1**	0.009	0.625	0.016	0.750	0.017	0.500	0.044	0.250	0.005	1.000
		**Line 6.2-Line 8.1**	0.008	0.800	0.009	0.600	0.008	0.600	0.007	0.800	0.011	0.600
		**Line 8.1-Line 6.2**	0.035	**<0.001**	0.017	0.400	0.026	0.300	0.049	0.100	0.051	**<0.001**
		**Line 6.2-Line 8.2**	0.020	0.625	0.021	0.375	0.009	0.875	0.007	1.000	0.012	0.250
		**Line 8.2-Line 6.2**	0.075	0.125	0.047	0.125	0.074	0.375	0.120	0.125	0.035	0.250
		**Line 8.1-Line 8.2**	0.021	**<0.001**	0.029	**<0.001**	0.019	0.375	0.022	0.250	0.010	0.625
		**Line 8.2-Line 8.1**	0.021	0.875	0.012	0.875	0.035	0.750	0.049	0.625	0.003	0.875
**Tomato**	**batch 1**	**Line 2.1-Line 2.2**	0.055	0.250	0.026	0.500	0.072	**<0.001**	0.173	0.250	0.053	0.750
		**Line 2.2-Line 2.1**	0.450	0.500	0.429	0.500	0.468	0.500	0.598	0.250	0.476	0.500

		**Line 2.1-Line 7.1**	0.016	0.400	0.026	0.200	0.040	0.600	0.028	0.600	0.050	**<0.001**
		**Line 7.1-Line 2.1**	0.093	0.600	0.072	0.600	0.030	0.600	0.063	0.600	0.046	1.000
		**Line 2.1-Line 7.2**	0.007	0.857	0.025	0.571	0.031	0.714	0.015	0.714	0.015	0.857
		**Line 7.2-Line 2.1**	0.026	0.286	0.028	0.429	0.021	0.714	0.022	0.857	0.016	0.714
		**Line 2.2-Line 7.1**	0.516	0.333	0.526	0.333	0.495	0.333	0.663	0.333	0.517	0.333
		**Line 7.1-Line 2.2**	0.048	0.667	0.058	0.333	0.029	0.667	0.170	0.333	0.067	0.333
		**Line 2.2-Line 7.2**	0.337	1.000	0.264	1.000	0.288	1.000	0.398	0.600	0.348	1.000
		**Line 7.2-Line 2.2**	0.025	1.000	0.030	0.800	0.026	0.800	0.022	0.800	0.020	0.800
		**Line 7.1-Line 7.2**	0.013	1.000	0.068	1.000	0.011	0.833	0.078	1.000	0.016	1.000
		**Line 7.2-Line 7.1**	0.054	0.167	0.058	0.333	0.027	0.500	0.059	0.167	0.052	0.167
		**Line 4.1-Line 4.2**	0.028	0.800	0.057	0.400	0.052	0.200	0.048	0.400	0.063	0.400
		**Line 4.2-Line 4.1**	0.390	1.000	0.450	1.000	0.445	1.000	0.459	1.000	0.437	1.000
		**Line 4.1-Line 5.1**	0.038	0.800	0.052	0.400	0.056	0.200	0.053	0.200	0.040	0.400
		**Line 5.1-Line 4.1**	0.549	0.600	0.615	0.200	0.580	0.200	0.582	0.200	0.550	0.600
		**Line 4.1-Line 5.2**	0.027	0.800	0.006	1.000	0.002	1.000	0.004	1.000	0.004	1.000
		**Line 5.2-Line 4.1**	0.489	0.600	0.489	1.000	0.517	1.000	0.517	0.600	0.489	1.000
		**Line 4.1-Line 6.1**	0.024	0.286	0.006	1.000	0.006	0.857	0.007	1.000	0.014	0.571
		**Line 6.1-Line 4.1**	0.014	1.000	0.029	0.429	0.037	0.286	0.061	0.143	0.026	0.286
		**Line 4.1-Line 6.2**	0.043	0.500	0.009	0.750	0.011	0.500	0.011	0.500	0.006	1.000
		**Line 6.2-Line 4.1**	0.021	1.000	0.013	0.500	0.011	0.750	0.010	0.750	0.015	0.750
		**Line 4.1-Line 8.1**	0.011	1.000	0.042	1.000	0.027	1.000	0.043	1.000	0.024	1.000
		**Line 8.1-Line 4.1**	0.239	0.333	0.252	0.333	0.117	0.333	0.252	0.333	0.129	0.333
		**Line 4.1-Line 8.2**	0.019	0.857	0.016	0.714	0.055	0.429	0.017	0.429	0.062	0.429
		**Line 8.2-Line 4.1**	0.158	0.143	0.085	0.143	0.183	**<0.001**	0.150	**<0.001**	0.192	0.143
		**Line 4.2-Line 5.1**	0.524	1.000	0.613	1.000	0.583	1.000	0.555	1.000	0.541	1.000
		**Line 5.1-Line 4.2**	0.524	1.000	0.613	1.000	0.583	1.000	0.555	1.000	0.541	1.000
		**Line 4.2-Line 5.2**	0.500	1.000	0.544	1.000	0.558	1.000	0.566	1.000	0.537	1.000
		**Line 5.2-Line 4.2**	0.500	1.000	0.544	1.000	0.558	1.000	0.566	1.000	0.537	1.000
		**Line 4.2-Line 6.1**	0.447	1.000	0.524	1.000	0.554	1.000	0.518	1.000	0.509	1.000
		**Line 6.1-Line 4.2**	0.019	1.000	0.023	1.000	0.007	1.000	0.013	0.750	0.018	0.750
		**Line 4.2-Line 6.2**	0.501	1.000	0.545	1.000	0.543	1.000	0.518	1.000	0.597	0.600
		**Line 6.2-Line 4.2**	0.014	1.000	0.014	0.400	0.007	0.800	0.011	0.800	0.012	0.800
		**Line 4.2-Line 8.1**	0.595	1.000	0.636	1.000	0.606	0.333	0.614	1.000	0.629	0.333
		**Line 8.1-Line 4.2**	0.019	1.000	0.018	1.000	0.055	0.333	0.020	1.000	0.067	0.333
		**Line 4.2-Line 8.2**	0.508	1.000	0.538	1.000	0.523	1.000	0.474	1.000	0.449	1.000
		**Line 8.2-Line 4.2**	0.058	0.500	0.021	0.750	0.020	0.750	0.050	0.500	0.046	0.750
		**Line 5.1-Line 5.2**	0.584	1.000	0.648	1.000	0.615	1.000	0.612	1.000	0.565	1.000
		**Line 5.2-Line 5.1**	0.584	1.000	0.648	1.000	0.615	1.000	0.612	1.000	0.565	1.000
		**Line 5.1-Line 6.1**	0.413	1.000	0.482	1.000	0.517	1.000	0.509	1.000	0.345	1.000
		**Line 6.1-Line 5.1**	0.019	0.750	0.032	0.500	0.011	0.750	0.022	0.500	0.029	1.000
		**Line 5.1-Line 6.2**	0.514	1.000	0.507	1.000	0.566	0.600	0.538	0.600	0.515	1.000
		**Line 6.2-Line 5.1**	0.015	0.800	0.009	1.000	0.009	0.600	0.012	1.000	0.012	0.800
		**Line 5.1-Line 8.1**	0.543	1.000	0.531	1.000	0.583	0.333	0.584	1.000	0.507	1.000
		**Line 8.1-Line 5.1**	0.025	1.000	0.046	0.667	0.036	0.667	0.022	1.000	0.023	0.667
		**Line 5.1-Line 8.2**	0.510	1.000	0.558	1.000	0.537	1.000	0.500	1.000	0.425	1.000
		**Line 8.2-Line 5.1**	0.028	1.000	0.018	0.750	0.022	1.000	0.028	1.000	0.032	0.750
		**Line 5.2-Line 6.1**	0.473	1.000	0.467	1.000	0.518	1.000	0.518	1.000	0.457	1.000
		**Line 6.1-Line 5.2**	0.025	0.500	0.018	0.750	0.016	0.750	0.028	0.250	0.022	0.500
		**Line 5.2-Line 6.2**	0.376	1.000	0.446	1.000	0.448	1.000	0.460	1.000	0.423	1.000
		**Line 6.2-Line 5.2**	0.020	1.000	0.026	0.800	0.023	0.800	0.023	0.800	0.016	1.000
		**Line 5.2-Line 8.1**	0.584	1.000	0.581	1.000	0.653	0.333	0.604	1.000	0.633	0.333
		**Line 8.1-Line 5.2**	0.020	1.000	0.021	1.000	0.103	0.333	0.019	1.000	0.071	0.333
		**Line 5.2-Line 8.2**	0.542	1.000	0.568	1.000	0.498	1.000	0.459	1.000	0.468	1.000
		**Line 8.2-Line 5.2**	0.050	0.750	0.029	0.500	0.039	0.750	0.070	0.500	0.049	0.500
		**Line 6.1-Line 6.2**	0.018	1.000	0.027	0.571	0.014	0.571	0.019	0.571	0.040	0.714
		**Line 6.2-Line 6.1**	0.076	0.286	0.051	0.143	0.026	0.286	0.038	0.429	0.083	0.286
		**Line 6.1-Line 8.1**	0.075	0.800	0.046	1.000	0.068	0.600	0.110	0.600	0.036	1.000
		**Line 8.1-Line 6.1**	0.256	0.200	0.121	0.800	0.119	0.800	0.274	0.400	0.116	0.600
		**Line 6.1-Line 8.2**	0.019	0.833	0.034	0.667	0.085	0.833	0.097	**<0.001**	0.063	0.833
		**Line 8.2-Line 6.1**	0.140	0.167	0.041	0.667	0.132	0.167	0.121	0.167	0.186	0.167
		**Line 6.2-Line 8.1**	0.069	0.333	0.035	**<0.001**	0.044	0.333	0.038	0.667	0.054	**<0.001**
		**Line 8.1-Line 6.2**	0.128	1.000	0.080	1.000	0.049	1.000	0.153	0.333	0.037	1.000
		**Line 6.2-Line 8.2**	0.039	0.286	0.024	0.571	0.073	0.286	0.041	**<0.001**	0.093	0.143
		**Line 8.2-Line 6.2**	0.129	0.143	0.039	0.429	0.122	0.286	0.061	0.429	0.127	0.286
		**Line 8.1-Line 8.2**	0.243	0.600	0.270	0.400	0.150	0.400	0.195	0.400	0.132	0.400
	**batch 2**	**Line 8.2-Line 8.1**	0.132	**<0.001**	0.119	0.600	0.101	0.200	0.097	**<0.001**	0.096	**<0.001**

Table S11: Model selection for fecundity (day 150) on the different plant species and for the different spider mite lines.

Model selection						
	df	AIC	LogLik	Chisq	Chi df	Pr(>Chisq)
Removal of non-significant variables						
Maximal model: fecundity ~ plant species * host spider mite line + (1|batch/island)						
Line * Plant Species	24	1578.6	-765.29	36.423	11	0.0001439***
Line + Plant Species	13	1593.0	-783.51			

Table S12: Model for longevity (day 150).

	coef	exp(coef)	se(coef)	z	p
Cucumber	-1.4067	2.45E-01	0.545	-2.58	9.8E-03
Tomato	1.4610	4.31E+00	0.304	4.80	1.6E-06
Line2	19.7995	3.97E+08	7130.753	0	1.00E+00
Line3	19.4612	2.83E+08	7130.753	0	1.00E+00
Line4	17.5856	4.34E+07	7130.753	0	1.00E+00
Line5	18.6972	1.32E+08	7130.753	0	1.00E+00
Line6	18.4765	1.06E+08	7130.753	0	1.00E+00
Line7	19.6781	3.52E+08	7130.753	0	1.00E+00
Line8	17.7892	5.30E+07	7130.753	0	1.00E+00

Table S13: Relationship between spider mite fitness proxies and genetic background based on microsatellites, using Procrustes and Mantel tests.

The distance matrices used for both the Procrustes and the Mantel test measure the differences between the genetic background of the different spider mite lines on their host plant (bean, cucumber, or tomato) and the differences between the (I) fecundity, (II) longevity, or (III) both fecundity and longevity at the last time point (150 days). For the distance table of the genetic background, the Bruvo distance metric (2004) was chosen, while for the performance table two different distance measures were used (i.e., Euclidian and Manhattan). The Mantel test was done with 9999 permutations based on the Pearson method.

	**Distance metric**	**Bean**		**Cucumber**		**Tomato**	
		m_{12}^2/R^2	*p-value*	m_{12}^2/R^2	*p-value*	m_{12}^2/R^2	*p-value*
I) Fecundity							
Procrustes	Euclidian	0.827	0.951	0.755	0.983	0.285	0.333
	Manhattan	0.827	0.951	0.755	0.983	0.285	0.333
Mantel	Euclidian	-0.620	0.914	-0.568	0.975	0.477	0.292
	Manhattan	-0.620	0.914	-0.568	0.975	0.477	0.292
II) Longevity							
Procrustes	Euclidian	0.721	0.903	0.769	0.800	0.458	0.339
	Manhattan	0.721	0.903	0.769	0.800	0.458	0.339
Mantel	Euclidian	-0.230	0.726	-0.542	0.900	0.165	0.417
	Manhattan	-0.230	0.726	-0.542	0.900	0.165	0.417
III) Fecundity and Longevity							
Procrustes	Euclidian	0.772	0.971	0.753	0.983	0.241	0.333
	Manhattan	0.749	0.993	0.742	0.983	0.199	0.333
Mantel	Euclidian	-0.631	0.943	-0.571	0.975	0.522	0.208
	Manhattan	-0.687	0.979	-0.602	0.983	0.541	0.208

Reference

Bruvo R, Michiels NK, D'Souza TG, Schulenberg H. A simple method for the calculation of microsatellite genotype distances irrespective of ploidy level. Mol Ecol 2004; 13: 2101–2106.

Table S14: Relationship between spider mite fitness proxies and microbiome community structure (only including the Rickettsiales order), using Procrustes and Mantel tests.

The distance matrices used for both the Procrustes and the Mantel test measure the differences between the microbiome composition of the different spider mite lines on their host plant (bean, cucumber, or tomato) and the differences between the fecundity, longevity, or both fecundity and longevity at the last time point (150 days). For the microbiome distance table, the unweighted UniFrac was chosen, while for the performance table two different distance measures were used (i.e., Euclidian and Manhattan). The Mantel test was done with 9999 permutations based on the Pearson method.

		Fecundity						Longevity						Fecundity and Longevity					
		bean		cucumber		tomato		bean		cucumber		tomato		bean		cucumber		tomato	
		st.	p-v.	st.	p-v.	st.	p-v.	st.	p-v.	st.	p-v.	st.	p-v.	st.	p-v.	st.	p-v.	st.	p-v.
Procrustes	Euclidian	0.74	0.635	0.53	0.078	0.58	0.243	0.71	0.494	0.76	0.581	0.38	0.089	0.69	0.622	0.53	0.077	0.52	0.221
		0.62	0.113	0.46	**0.042**	0.61	0.257	0.71	0.404	0.67	0.212	0.41	0.115	0.59	0.140	0.45	**0.041**	0.55	0.244
		0.67	0.275	0.50	0.069	0.62	0.256	0.71	0.378	0.71	0.306	0.47	0.114	0.63	0.279	0.50	0.066	0.56	0.233
		0.70	0.372	0.47	**0.049**	0.57	0.249	0.74	0.746	0.66	0.138	0.41	0.092	0.65	0.392	0.46	**0.044**	0.51	0.215
		0.73	0.510	0.40	**0.034**	0.66	0.292	0.74	0.511	0.62	0.146	0.42	0.103	0.69	0.540	0.39	**0.031**	0.61	0.256
	Manhattan	0.74	0.635	0.53	0.078	0.58	0.243	0.71	0.494	0.76	0.581	0.38	0.089	0.67	0.694	0.51	0.080	0.46	0.143
		0.62	0.113	0.46	**0.042**	0.61	0.257	0.71	0.404	0.67	0.212	0.41	0.115	0.57	0.161	0.43	**0.030**	0.49	0.229
		0.67	0.275	0.50	0.069	0.62	0.256	0.71	0.378	0.71	0.306	0.47	0.114	0.61	0.312	0.48	0.064	0.51	0.203
		0.70	0.372	0.47	**0.049**	0.57	0.249	0.74	0.746	0.66	0.138	0.41	0.092	0.63	0.455	0.44	**0.042**	0.45	0.165
		0.73	0.510	0.40	**0.034**	0.66	0.292	0.74	0.511	0.62	0.146	0.42	0.103	0.68	0.605	0.37	**0.031**	0.56	0.172
Mantel	Euclidian	-0.17	0.705	0.09	0.311	0.13	0.351	0.10	0.317	-0.04	0.505	0.47	0.074	-0.15	0.683	0.09	0.306	0.19	0.342
		0.23	0.120	0.21	0.167	0.09	0.368	-0.05	0.572	0.26	0.257	0.47	0.069	0.22	0.129	0.21	0.163	0.15	0.308
		0.13	0.233	0.15	0.241	0.08	0.386	0.05	0.367	0.09	0.405	0.38	0.103	0.13	0.225	0.16	0.238	0.14	0.342
		0.01	0.473	0.28	0.107	0.12	0.353	-0.01	0.524	0.18	0.143	0.45	0.075	0.01	0.475	0.28	0.102	0.18	0.350
		-0.09	0.598	0.35	0.084	0.09	0.356	0.01	0.455	0.37	0.095	0.45	0.089	-0.09	0.604	0.35	0.080	0.16	0.358
	Manhattan	-0.17	0.705	0.09	0.311	0.13	0.351	0.10	0.317	-0.04	0.505	0.47	0.074	-0.15	0.685	0.08	0.317	0.24	0.275
		0.25	0.120	0.21	0.167	0.09	0.368	-0.05	0.572	0.26	0.257	0.47	0.069	0.23	0.130	0.22	0.154	0.20	0.283
		0.13	0.233	0.15	0.241	0.08	0.386	0.05	0.367	0.09	0.405	0.38	0.103	0.14	0.207	0.16	0.236	0.18	0.281
		0.01	0.473	0.28	0.107	0.12	0.353	-0.01	0.524	0.18	0.143	0.45	0.075	0.00	0.481	0.29	0.095	0.23	0.290
		-0.09	0.598	0.35	0.084	0.09	0.356	0.01	0.455	0.37	0.095	0.45	0.089	-0.09	0.598	0.37	0.072	0.20	0.347
